# Competition between type I activin and BMP receptors for binding to ACVR2A regulates signaling to distinct Smad pathways

**DOI:** 10.1186/s12915-022-01252-z

**Published:** 2022-02-18

**Authors:** Szabina Szófia Szilágyi, Ayelet R. Amsalem-Zafran, Keren E. Shapira, Marcelo Ehrlich, Yoav I. Henis

**Affiliations:** 1 Department of Neurobiology, George S. Wise Faculty of Life Sciences, Tel Aviv University, 6997801 Tel Aviv, Israel; 2 Shmunis School of Biomedicine and Cancer Research, George S. Wise Faculty of Life Sciences, Tel Aviv University, 6997801 Tel Aviv, Israel

**Keywords:** Activin, BMP, Receptor interactions, Receptor competition, FRAP, Signaling

## Abstract

**Background:**

Activins and bone morphogenetic proteins (BMPs) play critical, sometimes opposing roles, in multiple physiological and pathological processes and diseases. They signal to distinct Smad branches; activins signal mainly to Smad2/3, while BMPs activate mainly Smad1/5/8. This gives rise to the possibility that competition between the different type I receptors through which activin and BMP signal for common type II receptors can provide a mechanism for fine-tuning the cellular response to activin/BMP stimuli. Among the transforming growth factor-β superfamily type II receptors, ACVR2A/B are highly promiscuous, due to their ability to interact with different type I receptors (e.g., ALK4 vs. ALK2/3/6) and with their respective ligands [activin A (ActA) vs. BMP9/2]. However, studies on complex formation between these full-length receptors situated at the plasma membrane, and especially on the potential competition between the different activin and BMP type I receptors for a common activin type II receptor, were lacking.

**Results:**

We employed a combination of IgG-mediated patching-immobilization of several type I receptors in the absence or presence of ligands with fluorescence recovery after photobleaching (FRAP) measurements on the lateral diffusion of an activin type II receptor, ACVR2A, to demonstrate the principle of competition between type I receptors for ACVR2. Our results show that ACVR2A can form stable heteromeric complexes with ALK4 (an activin type I receptor), as well as with several BMP type I receptors (ALK2/3/6). Of note, ALK4 and the BMP type I receptors competed for binding ACVR2A. To assess the implications of this competition for signaling output, we first validated that in our cell model system (U2OS cells), ACVR2/ALK4 transduce ActA signaling to Smad2/3, while BMP9 signaling to Smad1/5/8 employ ACVR2/ALK2 or ACVR2/ALK3. By combining ligand stimulation with overexpression of a competing type I receptor, we showed that differential complex formation of distinct type I receptors with a common type II receptor balances the signaling to the two Smad branches.

**Conclusions:**

Different type I receptors that signal to distinct Smad pathways (Smad2/3 vs. Smad1/5/8) compete for binding to common activin type II receptors. This provides a novel mechanism to balance signaling between Smad2/3 and Smad1/5/8.

**Supplementary Information:**

The online version contains supplementary material available at 10.1186/s12915-022-01252-z.

## Background

Activins and bone morphogenetic proteins (BMPs) belong to the transforming growth factor-β (TGF-β) superfamily, which is comprised of 33 cytokines in humans and has critical roles in multiple physiological and pathological processes [1–7]. These ligands signal via hetero-tetrameric complexes of type II and type I receptors with Ser/Thr-kinase activity [8, 9]. They signal via the canonical Smad pathways, as well as by non-Smad signaling pathways [10–13]. Smad signaling is initiated by ligand binding to type II and type I receptors, with the type II receptor phosphorylating and activating the type I receptor, which then phosphorylates/activates receptor-specific Smads. These complex with Smad4 translocate to the nucleus and activate or repress transcription of diverse target genes [1, 14–16]. In mammals, 7 type I (activin-like receptor kinases; ALK1-7) and 5 type II receptors function in a combinatorial fashion. The specificity for the Smad pathway is determined by the type I receptors; thus, ALK4/5/7 and ALK1/2/3/6 activate Smad2/3 and Smad1/5/8, respectively [16]. TGF-β and activin ligands signal mainly via Smad2/3 (employing ALK5 for TGF-β and ALK4/7 for activins), while BMPs activate Smad1/5/8 via ALK1/2/3/6 [15–17]. However, in some cases, TGF-β and activins were shown to signal also to Smad1/5/8 via ALK1 (for TGF-β) or ALK2 (for activins and TGF-β) [18–21], and BMPs may induce some signaling to Smad2/3 [22–24]. Of note, activin signaling by specific ALK2 mutants (which have partial constitutive activity) to Smad1/5/8 is of special importance for fibrodysplasia ossificans progressiva (FOP) [7, 25]. The importance of the balance between activation of the two distinct Smad pathways is underscored by the opposing roles of pSmad1/5/8 (BMP activation) and pSmad2/3 (TGF-β1 stimulation) formation in several cellular contexts, including glioma [26], myeloma [27, 28], and adipocyte differentiation [29]. Interestingly, multiple reports connect activin, BMP, and TGF-β signaling with acquisition of pro-tumorigenic features in osteosarcoma [30–33].

Signaling by activins and BMPs is highly promiscuous, since apart from signaling through ALK4/7, the activin type II receptors (ACVR2A and 2B) can interact also with several type I BMP receptors (ALK1/2/3/6), which can also form complexes with the type II BMP receptor, BMPRII [15, 16, 34, 35]. Thus, competition between different BMP ligands for distinct BMP receptor variants was proposed to regulate BMP signaling profiles [36]. Of note, competition of activin with BMP ligands for various receptor complexes was suggested to regulate the activation of Smad2/3 vs. Smad1/5/8 pathways [21, 28, 37, 38], and activin A (ActA) was reported to form non-signaling complexes with ALK2 bound to type II activin receptors [39]. Since the first essential step in TGF-β family signaling is formation of signaling type I/II receptor complexes, this emphasizes the need for quantitative measurements of type I/II heterocomplex formation at the surface of live cells, as these form the basis for the distinct binding of different ligands and for the combinatorial formation of specific receptor heterocomplexes. Such studies were conducted on the full-length TGF-β and BMP receptors by co-patching and patch/FRAP (fluorescence recovery after photobleaching) studies [8, 40–46], demonstrating that these receptors form both heteromeric (type I/II) and homomeric (I/I or II/II) complexes already in the absence of ligand, with ligand binding increasing mainly heterocomplex formation. Recent studies based on receptors with partial cytoplasmic domain fused to β-galactosidase enzyme fragments were able to measure ligand-mediated interactions of ACVR2A with ALK2, of ACVR2B with ALK5 and of BMPRII with ALK4 [39], but no information is available on the interactions of these receptors in the absence of ligand and of the full-length receptors. Interestingly, while the heterocomplexes of the type II TGF-β receptor (TβRII) with the type I receptor ALK5 were stable, the BMPRII heterocomplexes with ALK3 or ALK6 were dynamic, suggesting weaker interactions [8]. The TβRII/ALK5 heterotetra-meric structure was verified by X-ray studies on ALK5/TβRII ectodomains (ED) in complex with TGF-β3 or -β1 [47, 48]. Tetrameric receptor complex structures were also found for the crystals of the EDs of ACVR2B/ALK1 complexed with BMP9, and the EDs of ACVR2A or 2B with ALK3 and BMP2 [35, 49, 50], and recently for the EDs of ACVR2B with ALK5 and GDF11 [9]. However, the cytoplasmic domains of the receptors also contribute to type I/type II heterocomplex formation, as shown by the reduced interactions between ALK5/TβRII and between BMPRII/ALK3 or ALK6 following truncation of parts of the cytoplasmic domains [42, 46], and by the recent demonstration that the ALK2/BMPRII kinase domains form heterocomplexes via their C-terminal lobes [51].

Despite the high potential for combinatorial receptor complex formation among activin receptors, whose importance is emphasized by the dual nature of activins as pro- or anti-tumorigenic agents [52–55], studies on complex formation between full-length activin receptors at the cell surface in the absence and presence of ligands are lacking. We hypothesized that competition between multiple type I receptors for activin type II receptors could be a major mechanism that regulates the balance between signaling to the Smad2/3 and Smad1/5/8 branches. Here, we employed patch/FRAP studies to investigate the interactions between full-length ACVR2A and several type I receptors (ALK2/3/4/6). These studies demonstrated stable complex formation between ACVR2A and each type I receptor, which was enhanced by ActA for ACVR2A/ALK4 and ACVR2A/ALK2 complexes. Of note, they demonstrated competition between ALK4 and type I BMP receptors (ALK2/3/6) for binding ACVR2A. Studies on ActA signaling to Smad2/3 and BMP9 or BMP2 signaling to Smad1/5/8 in U2OS cells, along with siRNA knockdown of ACVR2A, ACVR2B, BMPRII, or the type I receptors, showed that (i) signaling to both Smad branches by ActA or BMP9 is mediated by ACVR2A/B and not BMPRII (with ALK4 and ActA to pSmad2/3, and with ALK2/3 and BMP9 to pSmad1/5/8); (ii) BMP2 signaling to the Smad1/5/8 pathway can be induced via ACVR2A/ALK3, as well as via BMPRII and several BMP type I receptors. Importantly, the balance between ACVR2 signaling to the two Smad branches was regulated by competition between the type I receptors. We propose a model in which the type II activin receptor population is the target for competition between ALK4 and BMP type I receptors, which determine whether the signaling will be to the Smad2/3 or to the Smad1/5/8 pathway.

## Results

### ACVR2A forms stable complexes with ALK4 and with BMP type I receptors

We assessed competition between multiple type I receptors for the activin type II receptor (ACVR2) through a combination of IgG-mediated patching-immobilization of several type I receptors in the absence or presence of ligands with FRAP measurements on the lateral diffusion of ACVR2A.

**Fig. 1:**
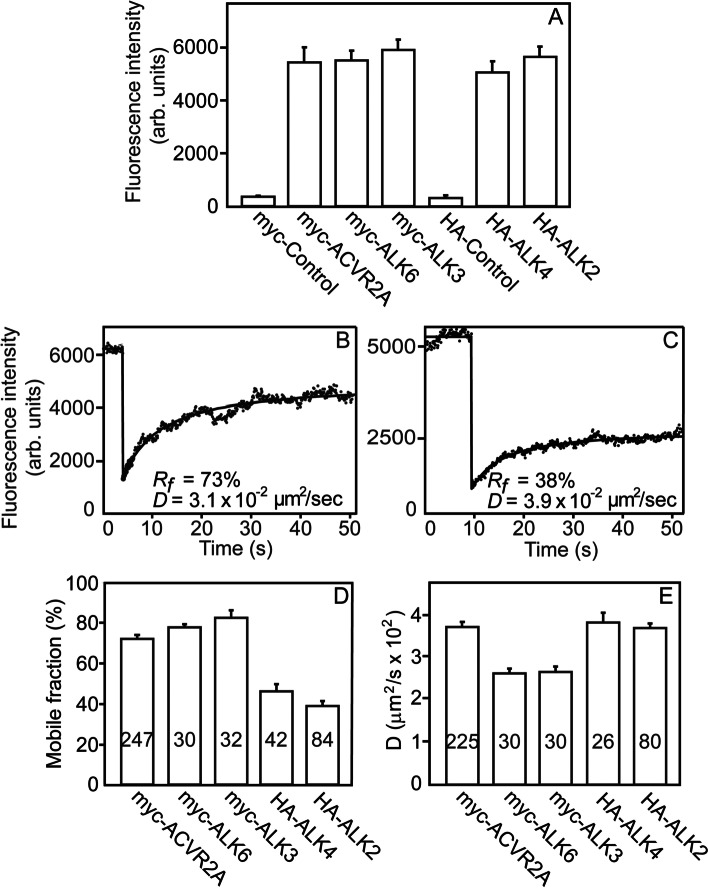
FRAP studies characterizing the lateral diffusion of various TGF-β-superfamily receptors. COS7 cells were transfected with an expression vector encoding myc- or HA-tagged receptor (myc-ACVR2A, myc-ALK6, myc-ALK3, HA-ALK4, or HA-ALK2), or empty vector (control). After 24 h, live cells were labeled by monovalent fluorescent Fab’ fragments as detailed under “Methods.” **a** Point-confocal measurements of the cell surface levels of the tagged receptors. Measurements were conducted as described under “Methods,” using the FRAP setup under identical non-bleaching conditions. Results are mean ± SEM of 30 independent measurements (each on a different cell) under each condition. “Control” designates cells transfected with empty vector (i.e., not expressing tagged receptors) incubated with αmyc (myc-Control) or αHA Fab’ (HA-Control) followed by secondary fluorescent Fab’ to yield background fluorescence levels. No significant differences were found between the expression levels of the various receptors, excluding the control samples (one-way ANOVA and Bonferroni post hoc test; *P* > 0.99). **b** A representative FRAP curve of the lateral diffusion of myc-ACVR2A. FRAP studies were conducted at 15 °C to minimize internalization. Solid lines are the best-fit of a nonlinear regression analysis to the lateral diffusion equation [56]. **c** A representative FRAP curve of HA-ALK4, which shows a lower mobile fraction (*R*_*f*_). **d**, **e** Average *R*_*f*_ and *D* values derived from multiple FRAP measurements. Bars are mean ± SEM; the number of measurements (each conducted on a different cell) is depicted on each bar. Some of these numbers are lower in panel D because FRAP curves yielding less than 20% recovery could be accurately analyzed only for *R*_*f*_

We first conducted FRAP studies to compare side-by-side the lateral diffusion coefficient (*D*) and mobile fraction (*R*_*f*_) of the receptors whose interactions were investigated in the current study. To this end, we expressed in COS7 cells [the system used to characterize the lateral diffusion and interactions of TGF-β and BMP receptors; 8, 40, 41-46] ACVR2A, ALK6, ALK3, ALK4, or ALK2 carrying an ED epitope tag (myc or HA). The cell surface receptors were labeled with monovalent Fab’ fragments (anti-tag followed by a fluorescent secondary Fab’) and subjected to FRAP studies. The cell surface expression levels of the tagged receptors, as measured by the fluorescence levels using point-confocal measurement of the fluorescence intensity by the FRAP instrumentation under identical labeling and illumination conditions (see “Methods”), were similar (Fig. 1 a). Figure 1 b, c depicts typical FRAP curves obtained for myc-ACVR2A and HA-ALK4, respectively; the average results derived from multiple FRAP experiments for each receptor are shown in Fig. 1 d, e. All these receptors exhibited lateral diffusion with *D* values typical of transmembrane proteins (2.5 to 4 × 10^−2^ μm^2^/s). The mobile fractions of ACVR2A, ALK6, and ALK3 were high (70–80%), similar to other TGF-β superfamily receptors [42, 45, 57–60], while ALK4 and ALK2 displayed significantly lower *R*_*f*_ values (40–50%). This suggests that a significant part of the population of the latter receptors interacts with cellular structures that are laterally immobile on this timescale.

To measure the mode (stable vs. transient) and extent of interactions between ACVR2A and the various type I receptors at the surface of live cells, we employed patch/FRAP [42, 58, 61]. In this method (for a schematic description, see Additional file 1: Fig. S1), one tagged receptor is patched and immobilized by crosslinking with a double layer of IgGs. The effect on the lateral diffusion of a coexpressed differently tagged receptor, labeled exclusively by monovalent Fab’ fragments, is measured by FRAP (see “Methods”). Depending on the mode and extent of the interactions, either *R*_*f*_ or *D* of the Fab’-labeled receptor can be reduced, depending on the dissociation/association rates of the receptor complex relative to the FRAP timescale. Complex lifetimes longer than the characteristic FRAP times (i.e., stable interactions) lead to a reduction in *R*_*f*_, since bleached Fab’-labeled receptors do not appreciably dissociate from the immobile clusters during the FRAP measurement. Conversely, short complex lifetimes (transient interactions) result in several association/dissociation cycles for each Fab’-labeled molecule during the FRAP measurement, reducing the effective *D* value without altering *R*_*f*_ [42, 61, 62]. Our previous studies indicated that TGF-β-superfamily receptors can interact in the absence of ligand, and these interactions may be enhanced by ligand [8, 42–45]. To determine which ligand concentration to use in such experiments, we characterized the dependence of pSmad formation by the ligands employed in the current study (ActA, BMP9 and BMP2) on the dose and time (Additional file 1: Figs. S2 and S3). Based on these data, we chose to test the effects of saturating amounts of the ligands, in order to ensure homogeneous ligand-bound receptor population.

**Fig. 2:**
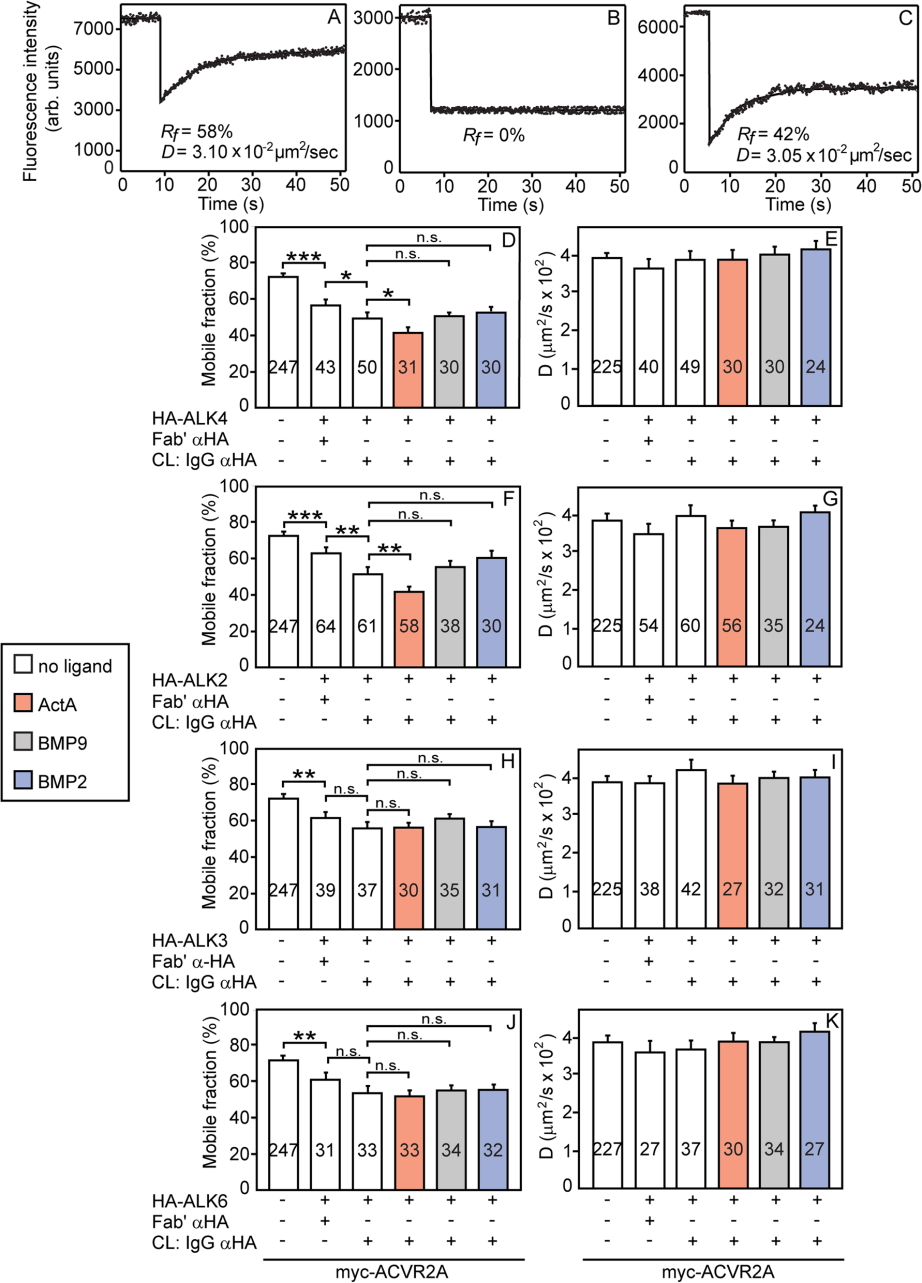
ACVR2A forms mutual heteromeric complexes with multiple type I receptors. COS7 cells were cotransfected with pairs of expression vectors encoding myc-ACVR2A along with an HA-tagged type I receptor (ALK4, ALK2, ALK3, or ALK6). After 24 h, live cells were subjected to the IgG-mediated patching/crosslinking (CL) protocol (“Methods”), resulting in the HA-type I receptor patched and labeled by Alexa 488-GαR IgG (designated “IgG αHA”), whereas myc-ACVR2A is labeled exclusively by monovalent Fab’ (with Alexa 546-GαM Fab’ as a secondary antibody). In control experiments without HA-type I receptor crosslinking, the IgG labeling of the HA tag was replaced by exclusive Fab’ labeling. Where indicated, ligand (4 nM ActA or BMP9, or 10 nM BMP2) was added during the last fluorescent labeling step for the FRAP experiment, and maintained throughout the measurement. FRAP studies were conducted as in Fig. 1. **a–c** Representative FRAP curves of myc-ACVR2A coexpressed with uncrosslinked (Fab’-labeled) HA-ALK4 (**a**), of HA-ALK4 immobilized by IgG crosslinking (**b**), and of myc-ACVR2A coexpressed with IgG-crosslinked HA-ALK4 (**c**). **d–k** Average *R*_*f*_ (**d**, **f**, **h**, **j**) and *D* values (**e**, **g**, **i**, **k**) depicting the effects of coexpression with various type I receptors and their crosslinking on the lateral diffusion of myc-ACVR2A. The bars depict the average values (mean ± SEM); the number of measurements (each conducted on a different cell) is shown on each bar. Some of these numbers are lower in the *D* value panels, since only *R*_*f*_ can be extracted from FRAP curves yielding less than 20% recovery. Asterisks indicate significant differences between the *R*_*f*_ values of the pairs indicated by brackets (*, *P* < 0.05; **, *P* < 5 × 10^−4^; ***, *P* < 10^−4^; one-way ANOVA and Bonferroni post hoc test. n.s. = non-significant). A similar analysis of the *D* values showed no significant differences (*P* > 0.2)

In the studies depicted in Fig. 2, myc-ACVR2A was coexpressed with an HA-tagged type I receptor, and the effects of the coexpression without and with IgG-mediated immobilization of the HA-tagged receptor on the lateral diffusion of myc-ACVR2A in the absence and presence of ligands were measured. Initially, we calibrated the coexpression conditions to ensure that expression of an HA-tagged type I receptor does not alter the cell surface level of myc-ACVR2A, and vice versa (Additional file 1: Fig. S4). Representative FRAP curves of the effects of HA-ALK4 (with or without immobilization) on the lateral diffusion of myc-ACVR2A (Fig. 2 a–c) are shown, along with average *D* and *R*_*f*_ values of multiple patch/FRAP experiments with each of the HA-type I receptors (Fig. 2 d–k). Coexpression with an uncrosslinked (Fab’-labeled) HA-type I receptor already induced a reduction in *R*_*f*_ of myc-ACVR2A relative to singly expressed ACVR2A, indicating that a subpopulation of ACVR2A interacts preferentially with slow-diffusing or immobile type I receptor molecules/clusters (compare the two leftmost bars in Fig. 2 d, f, h, and j). Immobilization of HA-ALK4 or HA-ALK2 by IgG crosslinking mediated a further significant reduction in *R*_*f*_ of the coexpressed myc-ACVR2A (Fig. 2 d, f), while the reduction in its *R*_*f*_ following immobilization of HA-ALK3 or HA-ALK6 was smaller and not significant (Fig. 2 h, j), indicating that the interactions of ALK4 and ALK2 with ACVR2A are stronger. In all cases, the *D* value of myc-ACVR2A was unaffected (Fig. 2 e, g, i, k). A reduction in *R*_*f*_ of a receptor (in this case, myc-ACVR2A) without an effect on its *D* value due to interactions with immobile HA-tagged receptors characterizes stable interactions between the HA- and myc-tagged receptor pairs on the time scale of the FRAP measurements [42, 58, 59, 61]. Interestingly, only the association of ALK4 and ALK2 with ACVR2A was enhanced by ligand (ActA), as indicated by the further ActA-mediated reduction in the *R*_*f*_ value of myc-ACVR2A in the presence of crosslinked HA-ALK4 or HA-ALK2 (Fig. 2 d, f), while BMP9 or BMP2 had no measurable effect on the interactions of any of the ACVR2A heterocomplexes studied (Fig. 2 d, f, h, j). As control, we probed whether an unrelated HA-tagged receptor (TβRII) interacts with myc-ACVR2A, and whether type I receptors from the activin and BMP Smad signaling arms (ALK4 and ALK2) interact with each other (Additional file 1: Fig. S5). Both controls came out negative.

### ALK4 and BMP type I receptors compete for binding ACVR2A

**Fig. 3:**
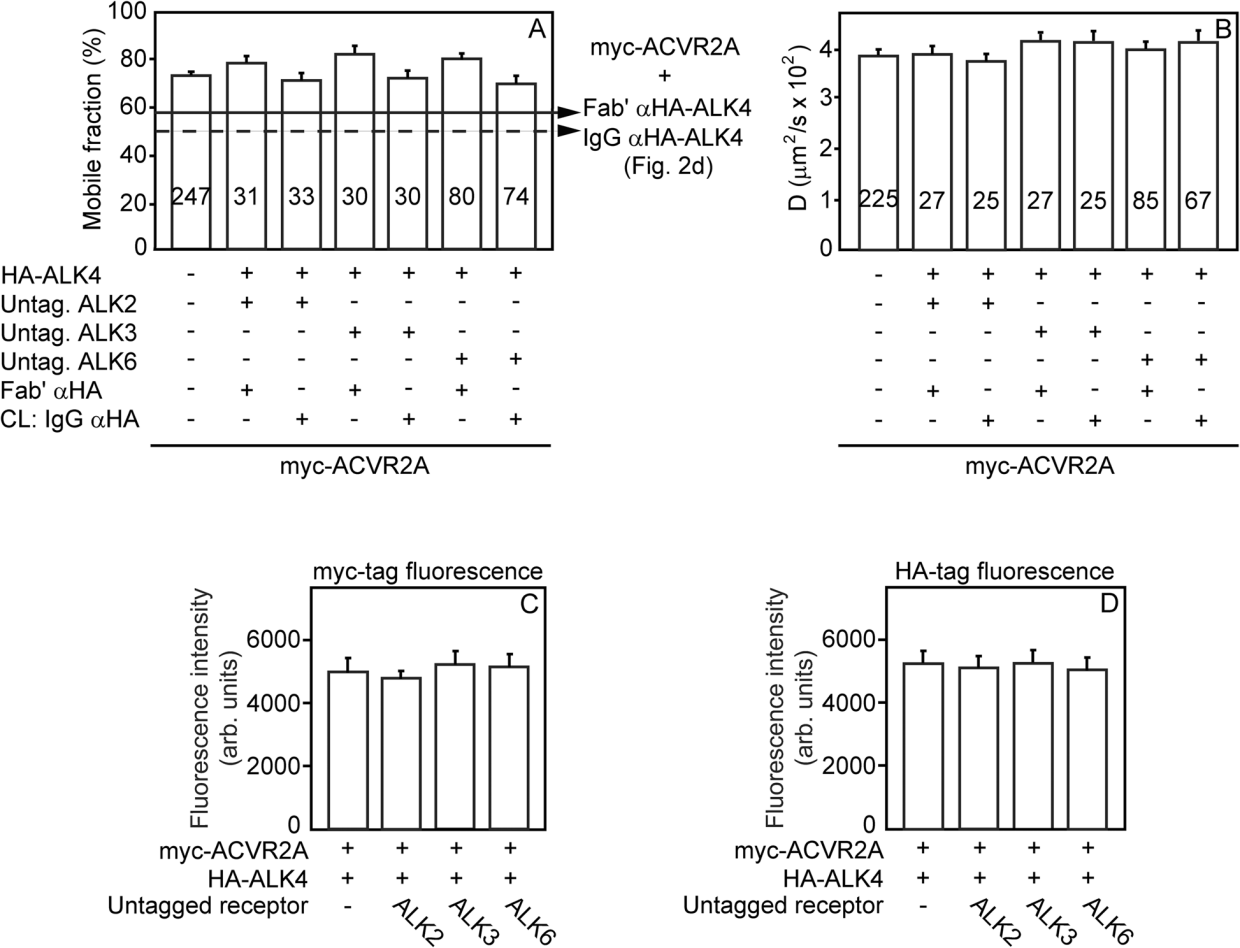
BMP type I receptors compete with ALK4 for binding to ACVR2A. Patch/FRAP studies were carried out on COS7 cells expressing myc-ACVR2A with HA-ALK4 (or empty vector). Where indicated, untagged ALK2, ALK3, or ALK6 was coexpressed with myc-ACVR2A and HA-ALK4. Where shown, HA-ALK4 was immobilized by IgG crosslinking as in Fig. 2. The lateral mobility of Fab’-labeled myc-ACVR2A was measured by FRAP. **a** Average *R*_*f*_ values; **b** Average *D* values. Bars are mean ± SEM; the number of measurements (on different cells) appears on each bar. The full and dashed lines depict the *R*_*f*_ values of myc-ACVR2A coexpressed with HA-ALK4 without (full line; taken from Fig. 2 d, second bar from the left, as indicated to the right of the panel) or with (dashed line; Fig. 2 d, third bar) IgG αHA crosslinking. No significant differences were found between *D* values of myc-ACVR2A upon coexpression with HA-ALK4 ±IgG crosslinking, or with or without coexpression with additional type I receptors (b) (one-way ANOVA with Bonferroni post hoc test; *P* > 0.7). The reduction in *R*_*f*_ of myc-ACVR2A upon immobilization of HA-ALK4 (Fig. 2 d) disappeared when untagged ALK2, ALK3, or ALK6 were coexpressed with myc-ACVR2A and HA-ALK4. Thus, the *R*_*f*_ values of myc-ACVR2A became similar to that of singly expressed myc-ACVR2A (**a**, leftmost bar; *P* > 0.05, one-way ANOVA and Bonferroni post hoc test), indicating that they compete with ALK4 for binding ACVR2A. **c**, **d** Point-confocal measurements of the expression levels of coexpressed myc-ACVR2A (**c**) and HA-ALK4 (**d**) with or without untagged ALK2, ALK3, or ALK6. The measurements were as described under “Methods.” Each bar represents mean ± SEM of 30 independent measurements. No significant differences were observed between the levels of either myc-ACVR2A or HA-ALK4 upon coexpression with one of the untagged type I receptors (one-way ANOVA and Bonferroni post hoc test; *P* > 0.99)

**Fig. 4:**
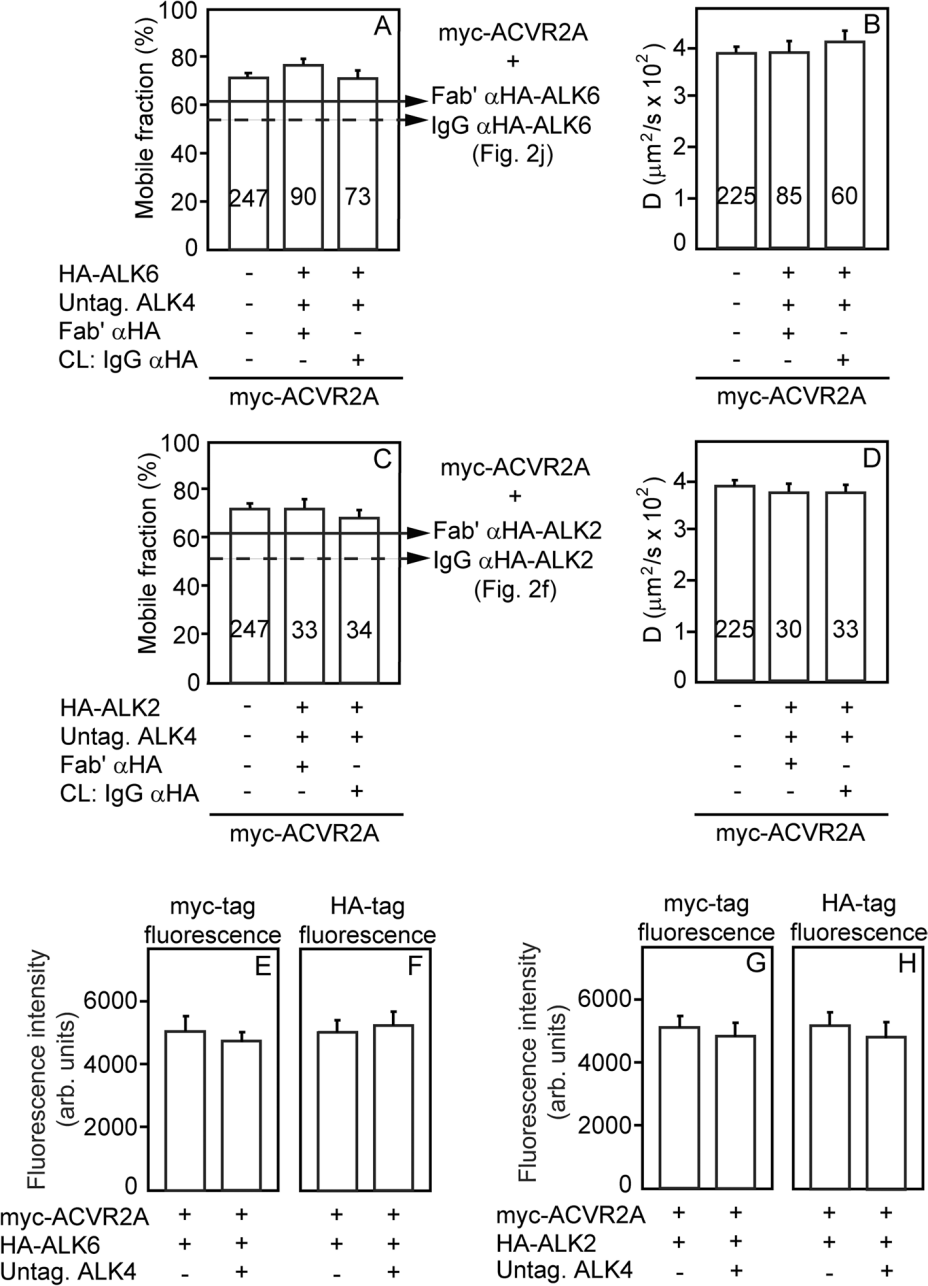
ALK4 competes with BMP type I receptors for binding ACVR2A. Experimental conditions were as in Fig. 3, with patch/FRAP measurements conducted on cells expressing myc-ACVR2A alone or together with HA-ALK6 or HA-ALK2 (instead of HA-ALK4). Where indicated, untagged ALK4 was coexpressed in addition to the myc- and HA-tagged receptors as a competitor. **a**, **c** Average *R*_*f*_ values; **b**, **d** Average *D* values. Bars are mean ± SEM; the number of measurements is depicted on each bar. The full and dashed lines show for comparison the *R*_*f*_ values of myc-ACVR2A coexpressed only with HA-ALK6 (**a**) or HA-ALK2 (**c**), as indicated to the right of the panels, without (full line) or with (dashed line) IgG αHA crosslinking of the indicated HA-tagged receptor (see Fig. 2f, j). No significant differences (one-way ANOVA with Bonferroni post hoc test; *P* > 0.5) were found between the *D* values under any condition (**b** and **d**). The reduction in *R*_*f*_ of myc-ACVR2A upon coexpression with HA-ALK6 or HA-ALK2 (with or without IgG crosslinking) disappeared following coexpression with untagged ALK4, and no significant differences were found from the *R*_*f*_ value of singly expressed myc-ACVR2A (*P* > 0.08, one-way ANOVA and Bonferroni post hoc test). These results indicate that ALK4 competes with HA-ALK2 or HA-ALK6 for binding myc-ACVR2A. **e–h** Point-confocal measurements of the expression levels of coexpressed receptors. **e** Level of myc-ACVR2A coexpressed with HA-ALK6, without or with untagged ALK4. **f** Level of HA-ALK6 with myc-ACVR2A alone or with untagged ALK4. **g** Level of myc-ACVR2A coexpressed with HA-ALK2 alone or with untagged ALK4. **h** Level of HA-ALK2 coexpressed with myc-ACVR2A alone or with untagged ALK4. Measurements were as described under “Methods.” Bars represents mean ± SEM of 30 independent measurements. No significant differences were observed between the levels of any receptor pairs compared (Student’s two-tailed *t* test; *P* > 0.5)

The results depicted in Fig. 2 demonstrate that all the type I receptors studied here (ALK4, ALK2, ALK3, and ALK6) can form complexes with ACVR2A, which are stable on the FRAP timescale. This led us to the hypothesis that ALK4 may compete with the type I BMP receptors for binding ACVR2A. To test this hypothesis, we performed patch/FRAP experiments to measure the effects of overexpression of one of these type I receptors (e.g., untagged ALK2) on the ability of an HA-tagged type I receptor (e.g., HA-ALK4) to interact with myc-ACVR2A. Cells were transfected with myc-ACVR2A alone, together with HA-ALK4, or together with both HA-ALK4 and an untagged BMP type I receptor. After IgG-mediated patching of HA-ALK4, all samples were subjected to patch/FRAP studies to determine the formation of heterocomplexes between myc-ACVR2A and HA-ALK4 (in the absence or presence of coexpressed untagged BMP type I receptor). As shown in Fig. 3 a, coexpression of each of the untagged BMP type I receptors completely abrogated the ability of coexpressed HA-ALK4 (with or without IgG crosslinking) to reduce *R*_*f*_ of myc-ACVR2A, and fully released ACVR2A to its original mobile fraction. This indicates that the overexpressed untagged type I receptor releases ACVR2A from its interactions with ALK4, as well as from interactions of the ACVR2A/ALK4 complexes with other immobile structures. Of note, no effects were observed on the lateral diffusion rate (*D*) of myc-ACVR2A (Fig. 3 b), in line with the stable nature of the myc-ACVR2A heterocomplexes on the FRAP timescale. Similarly, reciprocal effects show competition by the activin type I receptor ALK4 with type I BMP receptors (HA-ALK2 or HA-ALK6) for binding myc-ACVR2A (Fig. 4 a–d). Coexpression of untagged ALK4 markedly disrupted the ability of coexpressed and crosslinked HA-ALK2 or HA-ALK6 to reduce *R*_*f*_ of myc-ACVR2A (Fig. 4 a–d). In both Figs. 3 and 4, overexpression of an HA-tagged type I receptor did not alter significantly the expression level of myc-ACVR2A, and vice versa (Figs. 3c, d and 4 e–h). Control experiments with HA-TβRII, which does not bind to ACVR2A (Fig. S5), demonstrated that its coexpression (with or without IgG crosslinking) with untagged ALK4 and myc-ACVR2A does not release the interactions between the latter two receptors, indicating that HA-TβRII does not compete with ALK4 for binding to ACVR2A (Additional file 1: Fig. S6). Taken together, our findings demonstrate that ALK4 competes with the BMP type I receptors for binding to ACVR2A at the cell surface.

### Identification of receptors that mediate pSmad2/3 or pSmad1/5/8 formation upon stimulation with ActA, BMP9, or BMP2

**Fig. 5:**
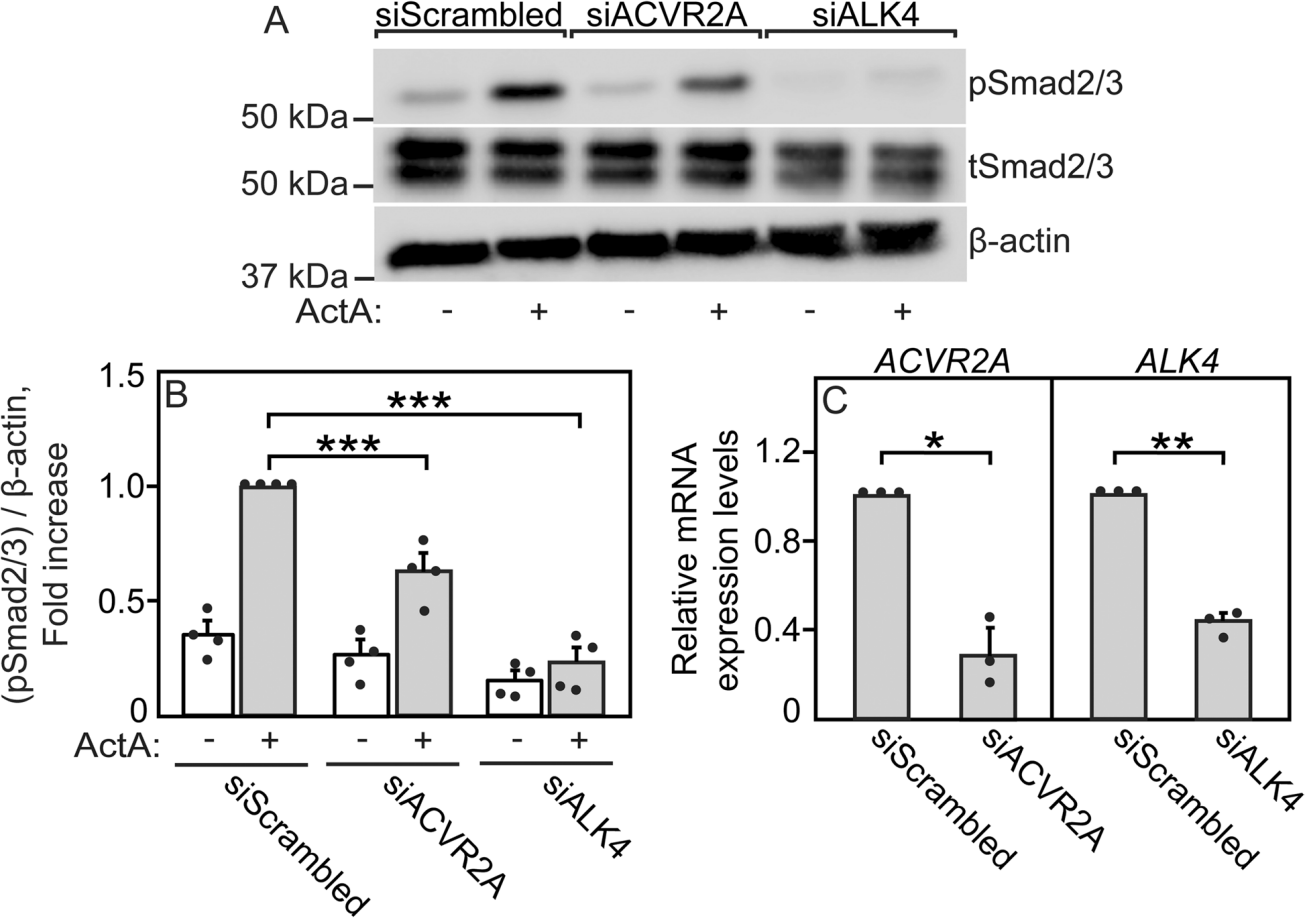
ActA signaling to Smad2/3 in U2OS cells is mediated via ALK4 and ACVR2A. Cells were transfected with siRNA to *ACVR2A*, *ALK4*, or scrambled siRNA (control). After 24 h, they were taken either for signaling studies (**a**, **b**) or for RT-qPCR determination of the mRNA levels of *ACVR2A* and *ALK4* (**c**). **a** A representative experiment. For the signaling studies, cells were starved (2 h, 1% serum) and stimulated (30 min, 37 °C) with ActA (4 nM), or left in starvation medium (control). Cells were lysed, subjected to SDS-PAGE, and immunoblotted for pSmad2/3, tSmad2/3 and β-actin. **b** Quantification of ActA signaling to Smad2/3. The bands were visualized by ECL and quantified by densitometry (see “Methods”). Data are mean ± SEM of the pSmad2/3 over β-actin ratio of 4 independent experiments. The value obtained for ActA-stimulated cells transfected with siScrambled RNA was taken as 1. pSmad2/3 formation in response to ActA in U2OS cells was almost fully abrogated by knockdown of *ALK4* and was significantly reduced by knocking down *ACVR2A* (or *ACVR2B*; Additional file 1: Fig. S7a, b). **c** RT-qPCR quantification of siRNA-mediated knockdown of *ALK4* or *ACVR2A*. Data were normalized using *GAPDH* as the housekeeping gene, taking the level of the respective mRNA in cells transfected with siScrambled as 1. The transcript expression levels of *ACVR2A* and *ALK4,* taken from the Cancer Cell Line Encyclopedia [65] are given in Additional file 2: Table S1. The results are the mean ± SEM of three independent experiments, each conducted in triplicate. Asterisks show significant differences between the pairs indicated by the brackets, using one-way ANOVA and Bonferroni post hoc test (for the signaling studies; panel **b**) or Student’s two-tailed *t* test (RT-qPCR, panel **c**). *, *P* < 0.02; **, *P* < 0.003; ***, *P* < 10^−4^

**Fig. 6:**
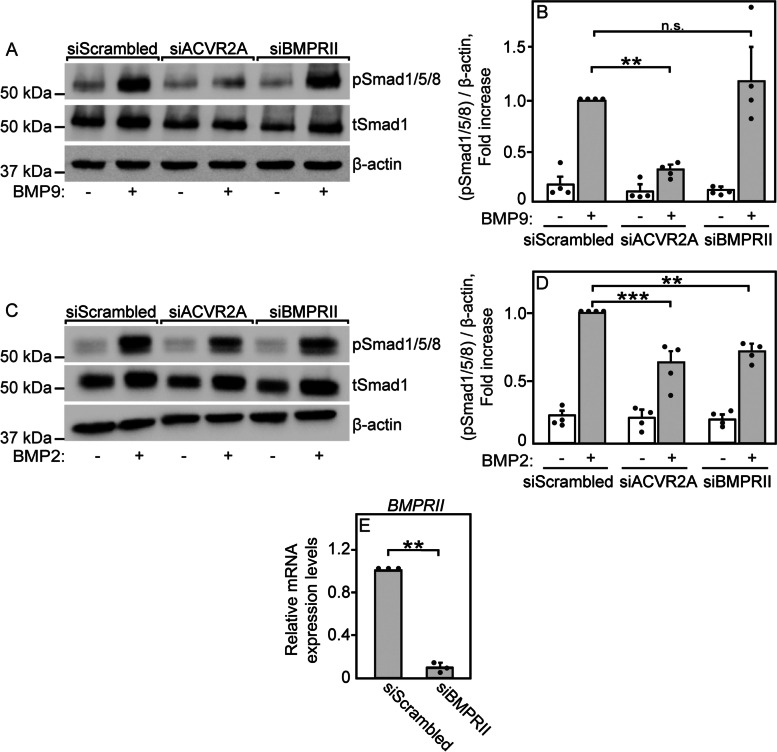
BMP9 signals to Smad 1/5/8 via ACVR2A, while BMP2 signals via ACVR2A and BMPRII. Cells were transfected with siRNA to *ACVR2A*, *BMPRII*, or siScrambled RNA (control). After 24 h, they were taken for signaling studies (**a–d**) or RT-qPCR determination of the mRNA levels of *ACVR2A* (Fig. 4 c) or *BMPRII* (**e**). **a**, **c** Representative signaling blots. Cells were starved (2 h, 1% serum), stimulated (30 min, 37 °C) or not (control) with 4 nM BMP9 (**a**) or 10 nM BMP2 (**c**), lysed, and subjected to SDS-PAGE followed by immunoblotting for pSmad1/5/8, tSmad1, and β-actin. **b**, **d** Quantification of BMP9 (**b**) and BMP2 (**d**) signaling to Smad1/5/8. Bands were visualized by ECL and quantified by densitometry. Data are mean ± SEM of the pSmad1/5/8 over β-actin ratio of 4 independent experiments. The value obtained for BMP-stimulated cells transfected with siScrambled RNA was taken as 1. pSmad1/5/8 formation in response to BMP9 (a, b) was markedly abrogated by si*ACVR2A* (and si*ACVR2B*; Additional file 1: Fig. S7c, d), but was unaffected by *BMPRII* siRNA. On the other hand, BMP2 signaling (c, d) to pSmad1/5/8 was reduced ~2-fold by siRNA to *ACVR2A* or *BMPRII*, but not by siRNA to *ACVR2B* (Fig. S7e, f). **e** RT-qPCR quantification of *BMPRII* (*ACVR2A* is shown in Fig. 5 c). Data were normalized to *GAPDH*, taking the *BMPRII* mRNA level in siScrambled cells as 1. The transcript expression level of *BMPRII* from the Cancer Cell Line Encyclopedia [65] is given in Additional file 2: Table S1. Results are mean ± SEM of three independent experiments, each conducted in triplicate. Asterisks indicate significant differences between the bracketed pairs (one-way ANOVA and Bonferroni post hoc test for signaling studies; **b**, **d**) or Student’s two-tailed *t* test for RT-qPCR (**e**). **, *P* < 0.001; ***, *P* < 10^−4^. n.s. = non-significant (*P* > 0.5)

**Fig. 7:**
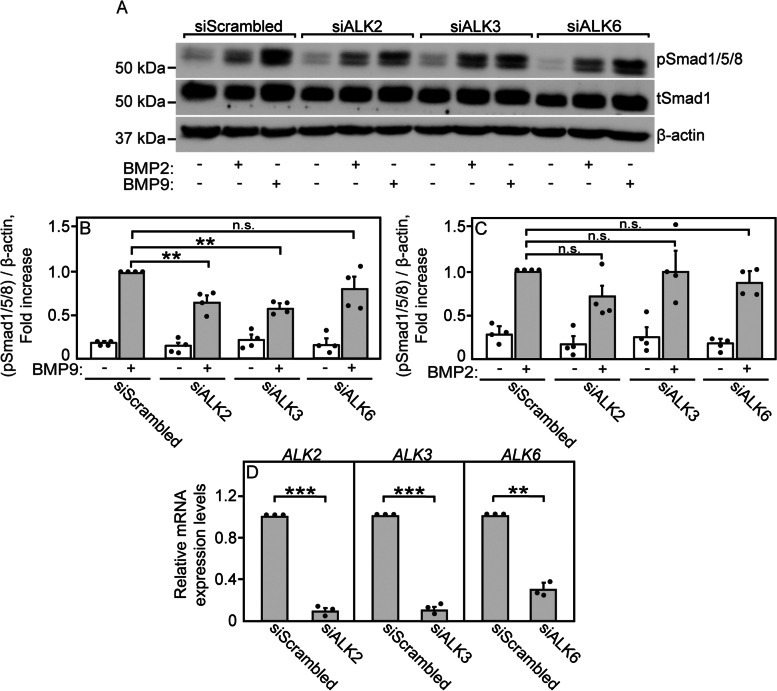
BMP9 and BMP2 signaling to Smad 1/5/8 depend differently on BMP type I receptors expression. Experimental conditions were as in Fig. 6, except that the transfected siRNAs were directed to *ALK2*, *ALK3*, *ALK6*. Scrambled siRNA served as control. **a** A typical blot of a signaling experiment. The experiments were conducted as in Fig. 6, and the blots were probed for pSmad1/5/8, tSmad1, and β-actin. **b**, **c** Quantification of BMP9 (**b**) or BMP2 (**c**) signaling to Smad1/5/8. Data are mean ± SEM of the pSmad1/5/8 over β-actin ratio of 4 independent experiments. The value obtained for BMP9- or BMP2-stimulated cells with siScrambled RNA was taken as 1. BMP9 signaling to pSmad1/5/8 was significantly reduced by knockdown of *ALK2* or *ALK3*, but not *ALK6*. On the other hand, BMP2 signaling to pSmad1/5/8 was unaffected by knocking down either one of these type I receptors, in line with BMP2 being able to signal via multiple such receptors. **d** RT-qPCR quantification of *ALK2*, *ALK3*, and *ALK6*. Data were normalized to *GAPDH* as in Figs. 5 and 6. The transcript expression levels of *ALK2*, *ALK3*, and *ALK6* from the Cancer Cell Line Encyclopedia [65] are given in Additional file 2: Table S1. Results are mean ± SEM of three independent experiments, each conducted in triplicate. Asterisks indicate significant differences between the pairs marked by the brackets, using one-way ANOVA and Bonferroni post hoc test for the signaling experiments (**b** and **c**; **, *P* < 0.002. n.s. = non-significant), and Student’s two-tailed *t* test for the RT-qPCR data (**d**; **, *P* < 0.005; ***, *P* < 5 × 10^−4^)

In order to correlate between receptor complex formation and signaling, we conducted signaling studies in U2OS cells, a human bone osteosarcoma cell line that expresses the various receptors employed in the current studies [39, 63, 64] (see Figs. 5 c, 6e, and 7d). The RPKM (reads per kilobase of transcript per million mapped reads) values for the mRNA expression levels of the TGF-β superfamily receptors in U2OS cells (Additional file 2: Table S1) were taken from the Cancer Cell Line Encyclopedia [65], employing cBioPortal [66].

To set the background for receptor competition studies on signaling, we first identified the type II and type I receptors that mediate ActA and BMP9 or BMP2 signaling in U2OS cells. To this end, we transfected the cells with siRNA to knock down a specific receptor (*ACVR2A*, *ACVR2B, BMPRII*, *ALK4*, *ALK2*, *ALK3*, or *ALK6*), using transfection with scrambled siRNA (siScrambled) as control. After starvation and stimulation with ligand (ActA, BMP9, or BMP2), the effects of the siRNA knockdown on signaling to form pSmad2/3 or pSmad1/5/8 were measured by Western blotting. ActA-mediated pSmad2/3 formation was significantly reduced by siRNA to *ACVR2A* (Fig. 5 a, b) or *ACVR2B* (Additional file 1: Fig. S7a, b) and was essentially abrogated by siRNA to *ALK4* (Fig. 5 a, b). Under these conditions, ActA did not induce measurable signaling to Smad1/5/8 (Additional file 1: Fig. S8), in line with the report that ActA/ACVR2/ALK2 complexes are non-signaling [39]. The efficacy of the siRNA knockdown of *ACVR2A*, *ACVR2B*, and *ALK4* was similar (60–80%; Fig. 5 c and Additional file 1: Fig. S7g). These results demonstrate that Smad2/3 phosphorylation following ActA stimulation in U2OS cells is mediated to a large degree via ACVR2A and ACVR2B, with ALK4 as the main type I receptor.

To explore the type II receptors that induce signaling to the Smad1/5/8 pathway by BMP ligands, we employed BMP9, which is known to signal mainly via ALK2 with ACVR2A/B, and BMP2, which is less selective and signals via multiple type II and type I receptors [27, 28]. BMP9-mediated pSmad1/5/8 formation was markedly reduced by siRNA to *ACVR2A* (Fig. 6 a, b) and to a lesser degree by siRNA to *ACVR2B* (Additional file 1: Fig. S7c, d), but was unaffected by siRNA to *BMPRII* (Fig. 6 a, b), although the siRNA knockdown of *BMPRII* was highly effective (Fig. 6 e). These findings suggest that in U2OS cells, BMP9 signaling to pSmad1/5/8 is induced mostly via ACVR2A. On the other hand, signaling to pSmad1/5/8 by BMP2 was only partially blocked (less than 50%) by either siRNA to *ACVR2A* or *BMPRII* (Fig. 6 c, d), suggesting that this ligand signals effectively through both receptors. Interestingly, siRNA to *ACVR2B* did not affect BMP2-mediated Smad1/5/8 formation (Additional file 1: Fig. S7e, f). The higher promiscuity of BMP2 relative to BMP9 [28] is apparent in experiments where various BMP type I receptors were knocked down (Fig. 7). Thus, BMP9 signaling to pSmad1/5/8 was inhibited to ~50% by siRNA to *ALK2* or *ALK3*, but not to *ALK6* (Fig. 7 a, b), although all three siRNAs exhibited effective knockdown (Fig. 7 d). Signaling of BMP9 via ALK2 and ALK3 is also supported by the ability of LDN212854 to suppress BMP9 signaling to pSmad1/5/8 already at 1.3 nM (Additional file 1: Fig. S9). LDN212854 inhibits mainly ALK2 (IC_50_ of 1.3 nM) and ALK1 (IC_50_ of ~3 nM) and to a lesser extent ALK3 [67]; however, U2OS cells express only traces of ALK1 (Additional file 2: Table S1), suggesting that the inhibition is mainly due to the effect on ALK2 (with a possible contribution of ALK3 inhibition). In contrast, BMP2 signaling to pSmad1/5/8 was not significantly reduced by siRNA to either *ALK2*, *ALK3*, or *ALK6* (Fig. 7 a, c), implying that it may employ multiple type I receptors. In conclusion, ACVR2A (as well as ACVR2B) appears to be a major participant in both ActA-mediated signaling to pSmad2/3 with ALK4, and in BMP9-mediated signaling to pSmad1/5/8 with ALK2 and ALK3. On the other hand, BMP2 signaling is more promiscuous. Thus, for the effects of competition on signaling, we proceeded with ActA and BMP9 stimulations, which are more specific, and with ACVR2A, on which the biophysical studies on receptor interactions were conducted.

### Competition between ALK4 and type I BMP receptors for ACVR2A regulates the signaling balance to the Smad2/3 and Smad1/5/8 pathways

After demonstrating that ALK4 and ALK2/3/6 compete for binding to ACVR2A, we turned to explore whether this competition is reflected in effects on signaling to the Smad pathways activated by these type I receptors (Smad2/3 by ALK4, and Smad1/5/8 by ALK2, ALK3, or ALK6). In these experiments, we chose conditions under which ACVR2 are the main type II receptors involved in signaling to both Smad pathways in U2OS cells: (i) ActA stimulation of pSmad2/3 formation, which is induced mainly via ACVR2A/B and ALK4 (Fig. 5 a, b and Additional file 1: Fig. S7a, b), and (ii) BMP9 stimulation of pSmad1/5/8 formation, induced mainly via ACVR2A in complex with ALK2 and/or with ALK3 (Figs. 6 a, b and 7 a, b). Throughout these experiments, the signaling is mediated via the endogenous receptors of U2OS cells, while the HA-tagged type I receptors serve as competitors. Of note, HA-tagged ALK3 and ALK6 were already shown to be active [44, 45, 68, 69]. In addition, we verified the signaling competence of HA-ALK4 to pSmad2/3 and HA-ALK2 to pSmad1/5/8 upon transfection of U2OS cells (Additional file 1: Fig. S10). Moreover, overexpression of HA-ALK2 did not induce ActA-mediated signaling to Smad1/5/8, and overexpression of HA-ALK4 did not enhance BMP9-mediated signaling to Smad2/3 (Additional file 1: Fig. S11).

**Fig. 8:**
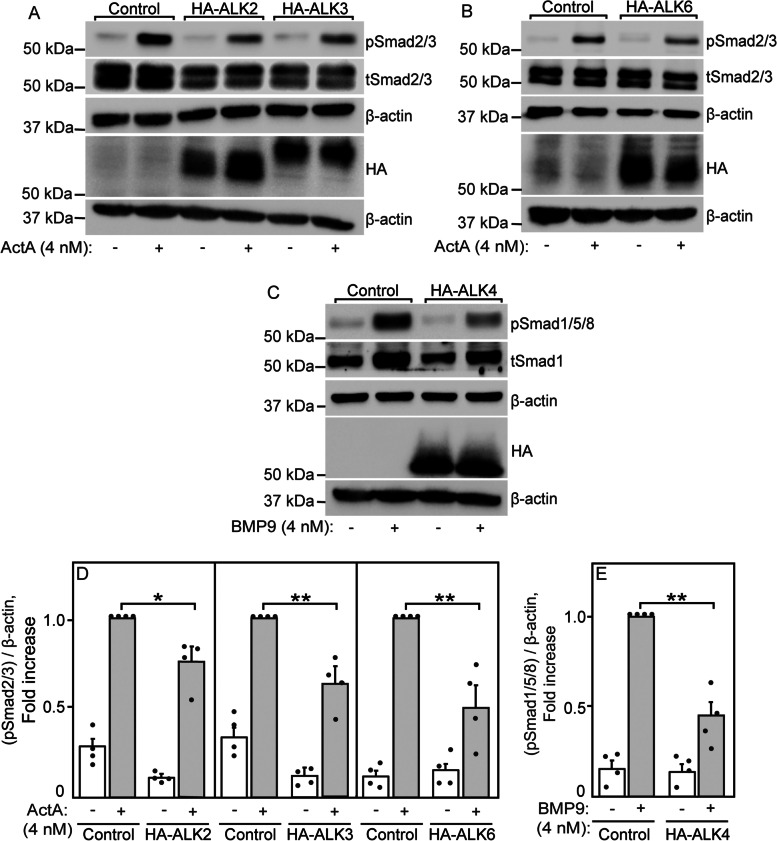
ALK4 and type I BMP receptors compete for signaling to distinct Smad pathways via ACVR2. U2OS cells were transfected with empty vector (control) or one of the type I receptors (HA-tagged ALK2, ALK3, ALK6, or ALK4). At 24 h post-transfection, they were starved (2 h, 1% serum) and stimulated (30 min, 37 °C) where indicated with 4 nM ActA or 4 nM BMP9. The cells were then subjected to lysis, SDS-PAGE and immunoblotting, probing for pSmad2/3, tSmad2/3 (for ActA signaling), pSmad1/5/8, tSmad1 (for BMP9 signaling), as well as for HA tag (using HA-7 αHA) and β-actin. **a**, **b** Representative blots of the effects of overexpression of ALK2, ALK3, or ALK6 on ActA signaling to pSmad2/3. **c** A representative blot showing the effect of ALK4 overexpression on BMP9 signaling to pSmad1/5/8. The expression of the various HA-tagged receptors was probed by blotting for the HA tag (**a–c**). **d**, **e** Quantification of ALK2/3/6 effects on ActA-mediated pSmad2/3 formation (**d**) and of ALK4 on BMP9-mediated pSmad1/5/8 formation (**e**). Data are mean ± SEM of the relevant pSmads over β-actin ratio of 4 independent experiments. The value obtained for control cells stimulated with ActA (**d**) or with BMP9 (**e**) was taken as 1. Asterisks indicate significant differences between the pairs marked by the brackets, using one-way ANOVA and Bonferroni post hoc test (*, *P* < 0.002; **, *P* <0.0001)

U2OS cells were transfected with empty vector (control) or with expression vectors for one of the HA-tagged type I receptors (ALK2, ALK3, ALK6, or ALK4). After starvation and stimulation with ligand (ActA or BMP9), the effects of overexpression of a competing type I receptor on ActA signaling to pSmad2/3 or on BMP9 signaling to pSmad1/5/8 were measured (Fig. 8). ActA signaling to pSmad2/3, which is induced in U2OS cells via ALK4, was inhibited significantly by either one of the BMP type I receptors tested, whose expression is demonstrated by immunoblotting of the HA tag (Fig. 8 a, b, d). These results are in line with the ability of all these type I receptors to bind to ACVR2A and to compete with ALK4 for binding to ACVR2A (Figs. 2 and 3). The effect of competition between distinct type I receptors on signaling is observed also on BMP9-mediated signaling to pSmad1/5/8, where overexpression of HA-ALK4 (shown by immunoblotting) significantly reduced BMP9-mediated formation of pSmad1/5/8 (Fig. 8 c, e). Of note, similar results were obtained when untagged type I receptors were employed as competitors (Additional file 1: Fig. S12), or when stimulation was by lower levels of ligands (Additional file 1: Fig. S13). These findings are in accord with the ability of ALK4 to compete with type I BMP receptors for binding to ACVR2A (Fig. 4). Since a potential alternative source for reducing the signaling output is by reduction of the level of the endogenous receptor (e.g., ALK4) by overexpression of, e.g., HA-ALK2 (and vice versa), we verified by RT-qPCR that the levels of the endogenous receptors were unchanged by transfection of a different HA-tagged receptor (no effect of HA-ALK2 expression on the mRNA level of ALK4, and vice versa; Additional file 1: Fig. S14). We conclude that all the type I BMP receptors studied here (ALK2, ALK3, or ALK6) compete with ALK4 for binding to ACVR2A and that this competition modulates the signaling between the two distinct Smad pathways (Smad2/3 *vs*. Smad1/5/8).

## Discussion

Activin and BMP receptors exhibit combinatorial interactions, resulting in an ability to form signaling complexes between different type II and type I receptors. Thus, the binding of multiple BMP ligands to BMP receptor variants was proposed to provide flexible regulation of BMP signaling profiles [36]. Importantly, this promiscuity enables regulation of the balance between signaling to pSmad2/3 (by activin via ACVR2A or 2B and ALK4/7) and to pSmad1/5/8 (by BMP via ACVR2A/B or BMPRII and ALK1/2/3/6) [15, 16, 34]. The binding of ligands to different complexes of these receptors has potential implications for multiple diseases, including glioma and myeloma [26–28], osteosarcoma [30–33] and FOP [7, 25, 27, 39]. While the effects of competition between different cytokines for the signaling receptors was extensively investigated [21, 28, 36–39], the competition between various type I receptors for binding to promiscuous activin type II receptors and its role in balancing signaling between the two Smad pathways was understudied.

To set the ground for patch/FRAP biophysical studies on the interactions between the full-length type II activin receptors (using ACVR2A to prove the concept) and different type I receptors, we first employed FRAP to quantify the lateral diffusion and mobile fractions of the receptors investigated in the current studies. As shown in Fig. 1, the *D* values measured for all these receptors were in the range of typical transmembrane proteins, close to that measured for other TGF-β superfamily receptors [8, 42, 45, 59, 60]. Interestingly, the *R*_*f*_ values of ALK4 and ALK2 were low relative to the other TGF-β superfamily receptors. It should be noted that the diffusion coefficient of membrane proteins or their complexes is only weakly (logarithmically) dependent on the mass of the protein embedded in the membrane [70], and thus is not expected to be altered merely by receptor complex formation. Therefore, lower *R*_*f*_ values suggest interactions of the receptor with membrane-associated structures which are immobile on the FRAP timescale. Such mobility-restricting interactions were shown to occur with the membrane-underlying cytoskeleton, the extracellular matrix, and structures such as clathrin-coated pits [57, 71–74].

To measure interactions between ACVR2A and type I receptors, we initially coexpressed HA-ALK4 or HA-ALK2 together with myc-ACVR2A, and measured the effect on the lateral diffusion of the latter (Fig. 2 d–g). For both of these type I receptors, their coexpression sufficed for a significant reduction in *R*_*f*_ of myc-ACVR2A. This most likely reflects interactions of ACVR2A with the immobile part of the ALK4 or ALK2 populations. Immobilizing the entire HA-ALK4 or HA-ALK2 populations by IgG crosslinking (Fig. 2 b) led to a further significant reduction in *R*_*f*_ of myc-ACVR2A. Addition of ActA (but not BMP9/2) resulted in a further reduction in *R*_*f*_, reflecting strengthening of the interactions between ACVR2A and ALK4 or ALK2. A somewhat different scenario was observed for the effects of coexpressing HA-ALK3 or HA-ALK6 with myc-ACVR2A. Here, mere coexpression of ALK3/6 was capable of inducing a mild reduction in *R*_*f*_ of myc-ACVR2A, suggesting that when in complex with ALK3 or ALK6, ACVR2A mobility is restrained by stronger interactions with immobile structures. However, further reduction in *R*_*f*_ of ACVR2A upon IgG crosslinking of these HA-tagged type I receptors was small and not statistically significant, suggesting weaker interactions with ACVR2A relative to those of ALK4 or ALK2, and there was no further effect on *R*_*f*_ upon addition of ligand (Fig. 2 h, j). Thus, comparison of the reduction in *R*_*f*_ of myc-ACVR2A under maximal conditions (coexpression with type I receptor/IgG immobilization/ActA) indicates a much stronger reduction induced by ALK4/2 (from 72 to ~40%), as compared to ALK3/6 (from 72 to ~55%). This indicates that the complexes of ACVR2A with ALK3/6 are weaker than those with ALK4/2. Of note, the *D* values of myc-ACVR2A were not significantly altered under any of the above conditions, indicative of the stable character of the interactions measured, as explained under “Results.” This differs from our former findings of transient (dynamic) interactions between BMPRII and ALK3 or ALK6 [45], while resembling the stable interactions measured between the type II TGF-β receptor and ALK5 [42]. Of note, although no direct interactions are detected between ALK2 and ALK4 (Additional file 1: Fig. S5e, f), one cannot exclude a contribution by possible interactions between other type I receptors, as well as modulation of heterologous receptor interactions by ligand heterodimers [75].

The finding that all the type I receptors tested could form complexes with ACVR2A raised the possibility that different type I receptors can compete for binding ACVR2A and that such competition can provide a mechanism to balance between signaling to the Smad2/3 and the Smad1/5/8 pathways. We devised an assay which tests the ability of coexpressing a third, untagged receptor, to disrupt the interactions between two other differently tagged receptors (e.g., myc-ACVR2A and HA-ALK4). Given the above-described stable nature of the ACVR2A complexes with the various type I receptors, effective competition is reflected in eliminating the reduction in *R*_*f*_ of myc-ACVR2A imposed by coexpression and crosslinking of the HA-tagged receptor (note the increase in *R*_*f*_ of myc-ACVR2A from the lower values shown by the horizontal arrows to values resembling singly expressed ACVR2A; Figs. 3 a and 4 a, c). It should be noted that all the untagged BMP type I receptors were able to compete with HA-ALK4 binding to myc-ACVR2A (i.e., increase the *R*_*f*_ value of myc-ACVR2A; Fig. 3), and untagged ALK4 effectively competed with HA-ALK2 or HA-ALK6 for association with ACVR2A (Fig. 4). Since patch/FRAP measures directly the interactions between the receptors at the cell surface (shown schematically in Additional file 1: Fig. S1), this indicates that the binding of the competing type I receptor to ACVR2A is sufficient for the inhibitory effect.

Competition for the formation of a given heterocomplex (e.g., ACVR2A/ALK4) is expected to reduce the signaling to the pathway activated by the specific complex (e.g., ActA-mediated pSmad2/3 formation), since the signal intensity should be proportional to the amount of activated signaling heterocomplexes. To test this scenario, we employed a two-pronged approach: siRNA-mediated knockdown identification of which receptors are involved in ActA-induced Smad2/3 or BMP9/2-mediated Smad1/5/8 activation in U2OS cells, followed by competition assays on signaling by these ligands to the two Smad pathways. Our initial experiments identified ACVR2A and ACVR2B as the major mediators of ActA (both ACVR2A and 2B) and BMP9 (mostly ACVR2A) induced Smad pathways in U2OS cells (Figs. 5 a, b, 6 a, b, and Additional file 1: Fig. S7a-d), while BMP2 signaling involved ACVR2A and BMPRII (Fig. 6 c, d) but not ACVR2B (Additional file 1: Fig. S7e, f). Moreover, ALK4 was the sole activator in ActA-mediated signaling to Smad2/3 (Fig. 5 a, b), and BMP9 stimulation of the Smad1/5/8 pathway involved the type I receptors ALK2 and ALK3 (Fig. 7 a, b), while BMP2 appeared to be promiscuous also for type I receptor usage (Fig. 7 a, c). For this reason, we designed the signaling competition assays with stimulation by ActA to Smad2/3 or by BMP9 to Smad1/5/8. In these assays, effective competition is demonstrated as a reduction in ActA-mediated pSmad2/3 formation upon expression of ALK2, ALK3, or ALK6, or as a reduction in BMP9-stimulated pSmad1/5/8 formation following expression of ALK4. Indeed, the signaling experiments confirmed the above predictions (Fig. 8). It should be noted that in these experiments the inhibition by the competing type I receptor is likely to be higher than reflected in the signaling experiments, because these biochemical studies reflect the signaling to the relevant Smad pathway in the entire cell population, and not only in the subpopulation transfected by the competing receptor.

**Fig. 9:**
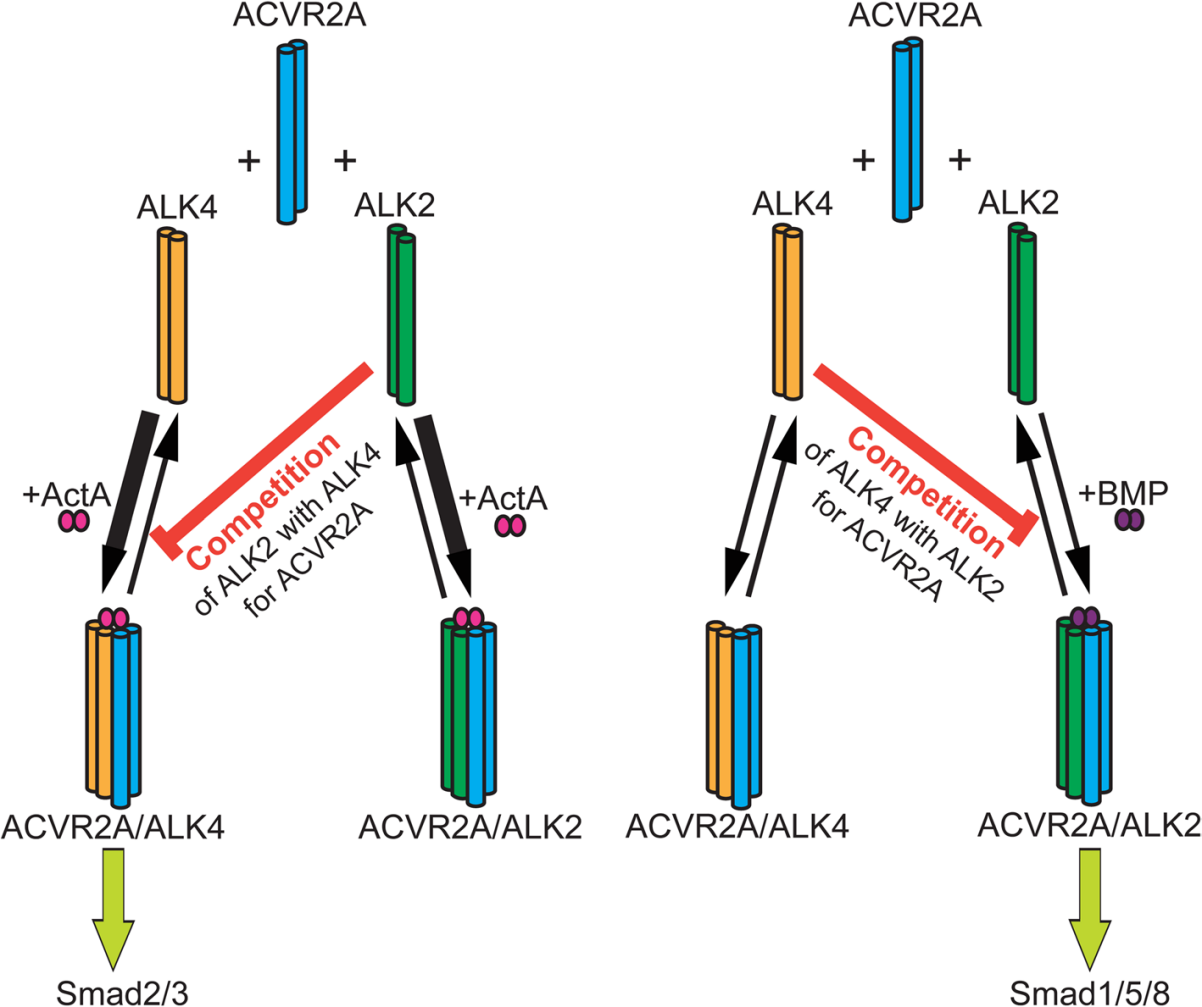
A model for the effect of type I receptor competition on the balance of signaling to Smad2/3 vs. Smad1/5/8. All receptors are designated as homodimers. Competition for ACVR2A (blue) is shown to demonstrate the principle. ACVR2A can form complexes with type I receptors that signal to Smad2/3 (e.g., ALK4, yellow) or Smad1/5/8 (e.g., ALK2, green). Excess of a type I receptor which signals to Smad1/5/8 (e.g., ALK2) can compete with ALK4 (which signals to Smad2/3) for binding to ACVR2A, and vice versa. In the case of ActA (left), ligand binding enhances heterocomplex formation with either ALK4 or ALK2 (thicker black arrows), while only the first induces pSmad2/3 formation. The competition by ALK2 inhibits ACVR2A/ALK4 complex formation, thus reducing ActA-mediated signaling to Smad2/3. In an analogous manner, binding of ALK4 to ACVR2A interferes with ACVR2A/ALK2 association (right), inhibiting BMP9-mediated signaling to Smad1/5/8

Based on the above findings, we propose a model (Fig. 9) where a promiscuous type II receptor (ACVR2A) can form heteromeric complexes with multiple type I receptors (in the model, we focus for simplicity on ALK2, which is a major inducer of pSmad1/5/8 by BMP9 stimulation in U2OS cells). Such complexes with ALK4 signal to the Smad2/3 pathway upon stimulation with ActA, while ACVR2A/ALK2 complexes signal to the Smad1/5/8 pathway following BMP9 stimulation. Since all BMP type I receptors in these cells (ALK2, ALK3, ALK6) can bind to ACVR2A, they compete with ALK4 for binding, thus inhibiting ACVR2A/ALK4 complex formation and ActA-mediated signaling to pSmad2/3. Similarly, ALK4 competition with ALK2 (or other BMP type I receptors) for binding to ACVR2A reduces ACVR2A/ALK2 complex formation and BMP9-induced signaling to pSmad1/5/8. Our results and the proposed model are in accord with the report that ActA forms non-signaling complexes with ACVR2-bound ALK2 [39], as shown also by the insignificant ActA signaling to Smad1/5/8 in U2OS cells (Additional file 1, Fig. S8). Moreover, we showed (Fig. 2 f) that ActA enhances ACVR2A/ALK2 interactions, a phenomenon that would enhance the competition-mediated inhibition by ALK2 to ActA-mediated Smad2/3 signaling. The current studies employ overexpression of specific receptors to provide a proof-of-principle for competition between different type I receptors for a given type II receptor as a mechanism for regulation of differential activation of Smad pathways. Providing that this principle holds also for receptors expressed at endogenous levels, such competition is expected to be affected by the relative expression levels of the receptors (and potentially co-receptors) in different cell types, and/or under different physiological conditions, providing another level of cell-specific regulation.

## Conclusions

In summary, we show for full-length receptors at the plasma membrane that the activin type II receptor (demonstrated here for ACVR2A) can form complexes with different type I receptors that signal either to Smad2/3 (ALK4) or to Smad1/5/8 (ALK2, ALK3, ALK6). We demonstrate that the different type I receptors compete for binding to ACVR2A and that this competition provides a mechanism that balances signaling between ActA-mediated, ALK4-dependent Smad2/3 signaling, and BMP-mediated ALK2 or ALK3-dependent signaling to Smad1/5/8. This mechanism provides the platform for which various ligands (e.g., activins, BMPs) can compete. Of note, such mechanisms may have important implications for several malignancies and/or diseases with cell differentiation-related etiologies.

## Methods

### Reagents

Recombinant human BMP2 (cat. #120-02C), BMP9 (cat. #120-07), and ActA (cat. #120-14P) were obtained from PeproTech (Rocky Hill, NJ). Media and cell culture reagents (fetal calf serum, L-glutamine, penicillin-streptomycin (25 and 40 μg/ml, respectively) and Hanks’ balanced salt solution (HBSS) were from Biological Industries Israel (Beit Haemek, Israel). Fatty acid-free bovine serum albumin (BSA) (fraction V; cat. #10-775-835-001) were obtained from Roche Diagnostics (Manheim, Germany). Phosphate-buffered saline, protease inhibitor cocktail (cat. #P8340), Na_3_VO_4_, LDN212854 (cat. #SML0965), and 4-(2-hydroxyethyl)-1-piperazineethanesulfonic acid (HEPES) were from Sigma-Aldrich (St. Louis, MO). Opti-MEM was from Gibco Life Technologies (Carlsbad, CA).

### Antibodies

Murine monoclonal anti-myc tag (αmyc, cat. #626802; RRID:AB_2148451) 9E10 IgG [76] and HA.11 rabbit polyclonal IgG to the HA tag (αHA, cat. #902302; RRID:AB_2565019) were from BioLegend (San Diego, CA). Murine monoclonal αHA IgG clone HA-7 (cat. #H3663; RRID:AB_262051) was from Sigma-Aldrich, and 12CA5 murine monoclonal αHA IgG (cat. #11666606001, RRID:AB_514506) was from Roche Diagnostics. Fab’ fragments were prepared from the murine 9E10 and 12CA5 IgGs as described [41]. Alexa Fluor (Alexa) 488-goat anti-rabbit (GαR) IgG (cat. #R37116; RRID:AB_2556544), Alexa 546-goat anti-mouse (GαM) F(ab')_2_ (cat. #A-11018; RRID:AB_2534085), and Alexa 488-GαR F(ab’)_2_ (cat. #A-11070; RRID:AB_142134) were from Invitrogen-Molecular Probes (Eugene, OR). Fluorescent F(ab′)_2_ were converted to monovalent Fab’ as described [40]. Normal goat γ-globulin (cat. #005-000-002; RRID:AB_2336984), peroxidase-conjugated GαM (cat. #115-035-062; RRID:AB_2338504), and GαR (cat. #111-035-144; RRID:AB_2307391) IgGs were from Jackson ImmunoResearch Laboratories (West Grove, PA). Rabbit antibodies to phospho (p) Smad1/5/8 (cat. #13820; RRID:AB_2493181), total (t) Smad1 (cat. #6944; RRID:AB_10858882), and pSmad2/3 (cat. #8828; RRID:AB_2631089) were from Cell Signaling (Danvers, MA). Murine IgG to tSmad2/3 (cat. #sc-133098; RRID:AB_2193048) was from Santa Cruz Biotechnology (Santa Cruz, CA), and mouse anti-β-actin (cat. #08691001; RRID:AB_2335127) from MP Biomedicals (Solon, OH).

### Plasmids and small interfering RNAs (siRNA)

Expression vectors encoding untagged human ALK2, N-terminally HA-tagged human ALK2 (with the HA tag, including a C-terminal two-glycine flexible linker, inserted by overlapping PCR after nucleotide 66) and untagged human ALK3 in pCMV5 were kindly donated by Prof. Petra Knaus (Freie Universität Berlin, Germany). Vectors encoding human ALK3 and murine ALK6 with extracellular myc or HA tags, as well as untagged ALK6, in pcDNA1 were described [44]. Human ACVR2A (in pcDNA3.1) was donated by Prof. Gerard Blobe (Duke University, Durham, NC); N-terminal myc tag was introduced by overlapping PCR after nucleotide 69 to generate myc-ACVR2A. N-terminal myc-tagged human ALK4 (cat. # LS-N16068) in pCMV3 was purchased from LSBio (Seattle, WA). Human ALK4 with C-terminal myc-DDK tags in pCMV6 (cat. #SC108895) was obtained from OriGene Technologies (Rockville, MD), and subcloned into pcDNA3.1 by PCR followed by restriction digest and re-ligation. A stop codon was introduced at nucleotide 1516 to delete the C-terminal tags to generate untagged ALK4. This was followed by insertion of N-terminal HA tag by overlapping PCR after nucleotide 72 to generate extracellularly tagged HA-ALK4. All constructs were verified by sequencing. ON-TARGETplus SMARTpool human siRNAs to *ACVR2A*, *BMPRII*, *ACVR1/ALK2*, *BMPR1A/ALK3*, *ACVR1B/ALK4*, and *BMPR1B/ALK6* as well as non-targeting pool (siScrambled) siRNA were purchased from Dharmacon (Lafayette, CO).

### Cell culture

COS7 (cat. #CRL-1651) and U2OS (cat. #HTB-96) cells (American Type Culture Collection, Manassas, VA) were grown in at 37 °C, 5% CO_2_ in Dulbecco’s modified Eagle’s medium supplemented with 10% FCS, penicillin, streptomycin, and L-glutamine as described earlier [44, 77]. The U2OS human cell line was authenticated by STR analysis at the Genomics Center of the Biomedical Core Facility, Technion, Haifa, Israel. All cells were routinely analyzed by reverse transcriptase-PCR (RT-PCR) for mycoplasma contamination and found to be clean.

### Transfection and siRNA-mediated knockdown

COS7 cells were transfected using TransIT-LT1 Mir2300 (cat. #MIR 2305, Mirus Bio, Madison, WI) according to manufacturer’s instructions. For Patch/FRAP experiments, cells grown on glass coverslips in 6-well plates were transfected with different combinations of vectors encoding myc- and/or HA-tagged (or untagged) receptor constructs. The amounts of the vectors (between 0.5 and 1 μg DNA) were adjusted to yield similar cell surface expression levels, determined by quantitative immunofluorescence as described by us earlier [45]. The total DNA level was complemented by empty vector to 2 μg. For signaling studies, U2OS cells were transfected with receptor constructs as above using jetPrime (cat. #114-15, Polyplus transfection, Illkirch, France), or with 50 nM final concentration of siRNA to the receptors detailed under plasmids and small interfering RNAs. For all experiments, cells were assayed 24–48 h post-transfection, as mentioned in the figure legends.

### Cell labeling and IgG-mediated patching for FRAP and patch/FRAP experiments

At 24 h post-transfection, COS7 cells transfected with various combinations of expression vectors for the above myc- and/or HA-tagged receptors were serum-starved (1% serum, 30 min, 37 °C), washed with cold HBSS containing 20 mM HEPES (pH 7.4) and 2% BSA (HBSS/HEPES/BSA), and blocked with normal goat γ-globulin (200 μg/ml, 30 min, 4 °C). For FRAP studies on singly expressed receptors, they were then labeled successively at 4 °C (to allow exclusive cell surface labeling) in HBSS/HEPES/BSA (45 min incubations) with (i) monovalent murine Fab’ αmyc or Fab’ of 12CA5 αHA (40 μg/ml); (ii) Alexa 546-Fab’ GαM (40 μg/ml). For patch/FRAP studies, they were labeled successively with (i) monovalent mouse Fab’ αmyc (40 μg/ml), alone or together with HA.11 rabbit αHA IgG (20 μg/ml) and (ii) Alexa 546-Fab’ GαM (40 μg/ml) alone or together with Alexa 488-IgG GαR (20 μg/ml). This protocol results in exclusive labeling of the myc-tagged receptor by monovalent Fab’, followed by measurement of its lateral diffusion by FRAP. In cells coexpressing an HA-tagged receptor, the protocol leads to crosslinking and immobilization of the HA tag by IgGs. In experiments with ligand, the ligands were added after starvation along with the normal goat γ-globulin and maintained at the same concentration (see figure legends) throughout the labeling steps and FRAP measurements.

### FRAP and Patch/FRAP

Cells co-expressing epitope-tagged receptors labeled fluorescently by Fab’ fragments (as described under cell labeling) were subjected to FRAP and patch/FRAP studies as described [42, 58]. FRAP studies were conducted at 15 °C, replacing samples after 20 min to minimize internalization. An argon-ion laser beam (Innova 70C, Coherent, Santa Clara, CA) was focused through a fluorescence microscope (Axioimager.D1; Carl Zeiss MicroImaging, Jena, Germany) to a Gaussian spot of 0.77 ± 0.03 μm (Planapochromat 63×/1.4 NA oil-immersion objective). After a brief measurement at monitoring intensity (528.7 nm, 1 μW), a 5-mW pulse (20 ms) bleached 60–75% of the fluorescence in the illuminated spot. Fluorescence recovery was followed by the monitoring beam. Values of *D* and *R*_*f*_ were derived by nonlinear regression analysis, fitting the FRAP curve to a lateral diffusion process [56]. Patch/FRAP studies were conducted analogously, except that IgG-mediated patching of an epitope-tagged receptor (described above) preceded the measurement [42, 61]. This measures the effects of immobilizing one receptor (HA-tagged) on the lateral diffusion of a coexpressed receptor (myc-tagged, labeled exclusively with monovalent Fab’). It detects complex formation between the receptors and distinguishes between transient and stable complexes [42, 58, 61].

### Point-confocal measurement of the cell surface levels of epitope-tagged receptors

Cells were transfected by myc- and/or HA-tagged receptors (alone or in various combinations) as described under “Transfection and siRNA-mediated knockdown.” After 24 h, the cell surface myc-tagged receptors (expressed alone or together with an HA-tagged receptor) were labeled at 4 °C as described under “Cell labeling” for FRAP studies on singly expressed receptors; the labeling employed a saturating concentration (40 μg/ml) of murine Fab’ αmyc, followed by 40 μg/ml Alexa 546-Fab’ GαM. HA-tagged receptors were labeled similarly on a separate sample, except that murine Fab’ of 12CA5 αHA replaced the Fab’ αmyc. This protocol enables to measure the levels of the tagged receptors at the plasma membrane under identical conditions (same laser excitation line and intensity, same microscope filters, same settings of the photomultiplier tube) [45]. The surface levels of the receptors were quantified by measuring the fluorescence intensity from a point-confocal spot by the FRAP apparatus under non-bleaching conditions at 15 °C, focusing the laser beam on the plasma membrane of a single cell at a time, replacing samples after 20 min to avoid internalization [45, 62].

### Western blot analysis

Then, 24–48 h post-transfection, U2OS cells were starved (1% serum, 2 h, 37 °C), and stimulated (or not) for 30 min with ligands at the concentrations mentioned in the figure legends. For experiments with the ALK2 inhibitor LDN212854, which is selective for ALK2 in preference to ALK3 (IC50 of 1.3 nM vs. 86 nM for ALK2 and ALK3, respectively) [67], the cells were incubated with the inhibitor (2.5 nM, 1 h) prior to ligand activation. Cells were lysed on ice (30 min) with lysis buffer (420 mM NaCl, 50 mM HEPES, 5 mM EDTA, 1% NP-40, 3 mM dithiothreitol, protease inhibitor cocktail and 0.1 mM Na_3_VO_4_). After low-speed centrifugation, the lysates were subjected to SDS-PAGE (10% polyacrylamide) followed by immunoblotting as described [78]. The blots were probed (12 h, 4 °C) by rabbit antibodies to pSmad1/5/8 (1:1000), pSmad2/3 (1:1000), or tSmad1 (1:1000), or by murine antibodies to tSmad2/3 (1:5000), αHA (the HA-7 antibody; 1:1000), or β-actin (1:50,000), followed by peroxidase-GαR or -GαM IgG (1:5000, 1 h). HA-tagged receptors were blotted by HA-7 αHA IgG (1:1000) followed by peroxidase-GαM IgG. The bands were visualized by enhanced chemiluminescence (ECL) using Clarity ECL substrate (cat. #1705060, Bio-Rad, Hercules, CA), recorded using ChemiDoc Touch imaging system (Bio-Rad) and quantified by Image Lab software (Bio-Rad).

### Real-time quantitative reverse transcriptase-PCR (RT-qPCR)

U2OS cells grown in 6-well plates were subjected to siRNA transfection followed by total RNA isolation using EZ-RNA kit (cat. #20-400-100, Biological Industries Israel) according to the manufacturer’s instructions. RNA was reverse transcribed to cDNA using Verso cDNA Synthesis Kit (cat. #AB-1453-B, Thermo Fisher Scientific). The mRNA levels of endogenous *ALK2, ALK3, ALK4, ALK6, ACVR2A,* and *BMPRII* were determined in triplicate by RT-qPCR using KAPA SYBR FAST ABI Prism qPCR kit (cat. #KK-KK4604, Kapa Biosystems-Roche, Wilmington, MA), and quantified with Applied Biosystems 7300 Real-Time PCR System Software (Thermo Fisher Scientific). Relative mRNA expression values were calculated based on the comparative threshold cycle (C_T_) method [79], normalizing the data to GAPDH. The sequences of the primers used for each receptor are listed in Supplementary Table S1.

### Statistical analysis

Statistical significance was analyzed by Prism9 (GraphPad Software, San Diego, CA). The significance of differences between multiple data sets was calculated using one-way ANOVA followed by post hoc Bonferroni test, and Student’s *t* test was used to calculate the difference between two groups, as described in the figure legends. Data are presented as mean ± SEM, along with the number of independent measurements (given within each figure or figure legend). A *P*-value lower than 0.05 was considered statistically significant.

## Supplementary Information

**Additional file 1: Figure S1.** Principle of the patch-FRAP method. **Figure S2.** Concentration dependence of Smad activation by ActA, BMP9 or BMP2 in U2OS cells. **Figure S3.** Smad activation as a function of time by ActA, BMP9 or BMP2 in U2OS cells. **Figure S4.** myc-ACVR2A cell surface levels are unaffected by coexpression of HA-type I receptors, and vice versa. **Figure S5.** Patch/FRAP studies do not detect interactions between myc-ACVR2A/HA-TβRII or myc-ALK4/HA-ALK2. **Figure S6.** HA-TβRII does not compete with ALK4 for binding myc-ACVR2A. **Figure S7.** ACVR2B signaling to Smad2/3 or Smad1/5/8 in U2OS cells. **Figure S8.** ActA does not induce significant signaling to Smad1/5/8 in U2OS cells. **Figure S9.** The ALK2/3 inhibitor LDN212854 inhibits BMP9-mediated pSmad1/5/8 formation in U2OS cells. **Figure S10.** Signaling activity of HA-ALK2 and HA-ALK4. **Figure S11.** HA-ALK2 and HA-ALK4 don’t promote ActA signaling to Smad1/5/8 or BMP9 signaling to Smad2/3, respectively. **Figure S12.** Untagged ALK4 and type I BMP receptors compete for signaling to Smads via ACVR2. **Figure S13.** Signaling competition by ALK4 and type I BMP receptors occurs also at lower ligand concentrations. **Figure S14.** Endogenous *ALK2* mRNA levels are not affected by overexpression of *ALK4* and *vice versa*.

**Additional file 2: Table S1.** Expression of TGF-β superfamily receptors in U2OS cells. **Table S2.** Sequences of the primer pairs used for RT-qPCR of each receptor gene.

**Additional file 3.** Original uncropped Western blots, PDF file.

## Data Availability

The data supporting the findings of this study are available within the article and its supplementary materials. All other supporting data are available from the corresponding authors on reasonable request.

## Abbreviations

ActA: Activin A
ACVR2A: Activin receptor type 2A
ACVR2B: Activin receptor type 2B
ALK1-7: Activin-like kinases 1 to 7
BMP: Bone morphogenetic protein
BMPRII: Type II BMP receptor
BSA: Bovine serum albumin
*D*: Lateral diffusion coefficient
ECL: Enhanced chemiluminescence
ED: Ectodomain
FOP: Fibrodysplasia ossificans progressiva
FRAP: Fluorescence recovery after photobleaching
GαM: Goat anti-mouse
GαR: Goat anti-rabbit
HBSS: Hank’s balanced salt solution
HEPES: 4-(2-Hydroxyethyl)-1-piperazineethanesulfonic acid
*R*_*f*_: Mobile fraction
RPKM: Reads per kilobase of transcript per million mapped reads
TGF-β: Transforming growth factor-β
TβRII: Type II TGF-β receptor
