# Supplementary material for: Competition between type I activin and BMP receptors for binding to ACVR2A regulates signaling to distinct Smad pathways
Source: BMC Biol. 2022 Feb 18;20:50. doi: 10.1186/s12915-022-01252-z (PMC8855587; doi:10.1186/s12915-022-01252-z)
Supplement: Supplementary file 1 — Additional file 1: Figure S1. Principle of the patch-FRAP method. Figure S2. Concentration dependence of Smad activation by ActA, BMP9 or BMP2 in U2OS cells. Figure S3. Smad activation as a function of time by ActA, BMP9 or BMP2 in U2OS cells. Figure S4. myc-ACVR2A cell surface levels are unaffected by coexpression of HA-type I receptors, and vice versa. Figure S5. Patch/FRAP studies do not detect interactions between myc-ACVR2A/HA-TβRII or myc-ALK4/HA-ALK2. Figure S6. HA-TβRII does not compete with ALK4 for binding myc-ACVR2A. Figure S7. ACVR2B signaling to Smad2/3 or Smad1/5/8 in U2OS cells. Figure S8. ActA does not induce significant signaling to Smad1/5/8 in U2OS cells. Figure S9. The ALK2/3 inhibitor LDN212854 inhibits BMP9-mediated pSmad1/5/8 formation in U2OS cells. Figure S10. Signaling activity of HA-ALK2 and HA-ALK4. Figure S11. HA-ALK2 and HA-ALK4 don’t promote ActA signaling to Smad1/5/8 or BMP9 signaling to Smad2/3, respectively. Figure S12. Untagged ALK4 and type I BMP receptors compete for signaling to Smads via ACVR2. Figure S13. Signaling competition by ALK4 and type I BMP receptors occurs also at lower ligand concentrations. Figure S14. Endogenous ALK2 mRNA levels are not affected by overexpression of ALK4 and vice versa. [file 12915_2022_1252_MOESM1_ESM.pdf]

Additional file 1: Supplementary figures S1 to S14

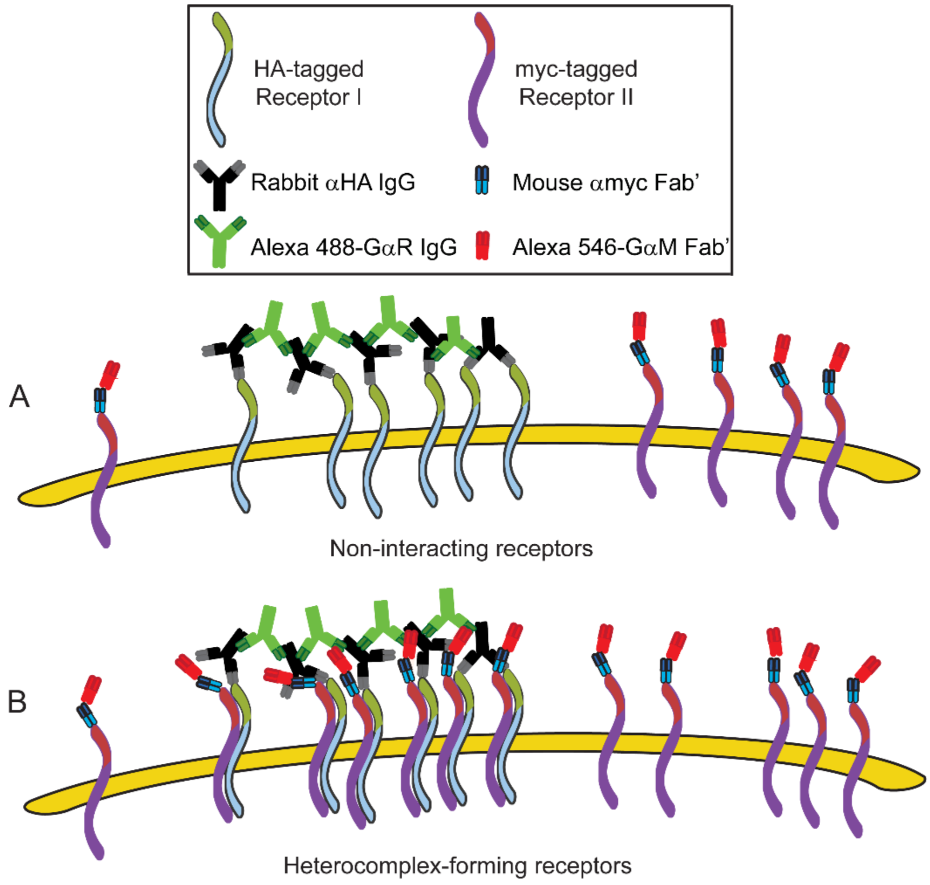

Fig. S1 Principle of the patch-FRAP method. In this method [42], two receptors, HA-tagged receptor I and myc-tagged receptor II (standing in most experiments in the current study for an HA-tagged type I receptor and myc-ACVR2A) with different extracellular epitope tags (HA and myc, designated by green and red extracellular domains, respectively) are coexpressed. The HA-receptor I (*e.g.*, HA-ALK4) cell surface population is labeled at 4 °C (to minimize internalization) by a double layer of IgGs, using rabbit αHA IgG primary antibody followed by Alexa 488-GαR IgG (green fluorescence) to crosslink and immobilize HA-receptor I. In parallel, the myc-tagged receptor II (*e.g.*, myc-ACVR2A) population is labeled by monovalent Fab' fragments from another species (murine αmyc Fab') followed by Alexa 546-GαM Fab' (red fluorescence). This protocol leads to immobilization of the IgG-crosslinked HA-receptor I, followed by measurement of the mobility of the red Fab'-labeled myc-receptor II by FRAP. **a** Non-interacting receptors. The IgG-crosslinked (green secondary IgGs) HA-receptor I (*e.g.*, HA-ALK4) population is immobilized; the lateral diffusion (both R_f and D) of the red Fab'-labeled myc-receptor II (*e.g.*, myc-ACVR2A is unaffected, as they do not bind to the IgG-patched receptors. **b** Heterocomplex-forming receptors. Part of the Fab'-labeled myc-receptor II population binds to the IgG-immobilized HA-receptor I. If the heterocomplexes are stable on the FRAP timescale, the bound Fab'-labeled receptors do not diffuse; thus, their R_f will be reduced. If the heterocomplexes are transient on the timescale of the FRAP measurement, D (rather than R_f) of the Fab'-labeled receptors (*e.g.*, myc-ACVR2A) will be reduced, because they would spend part of the measurement time bound to the immobilized crosslinked HA-receptor I, being free to diffuse during the dissociation cycle [42].

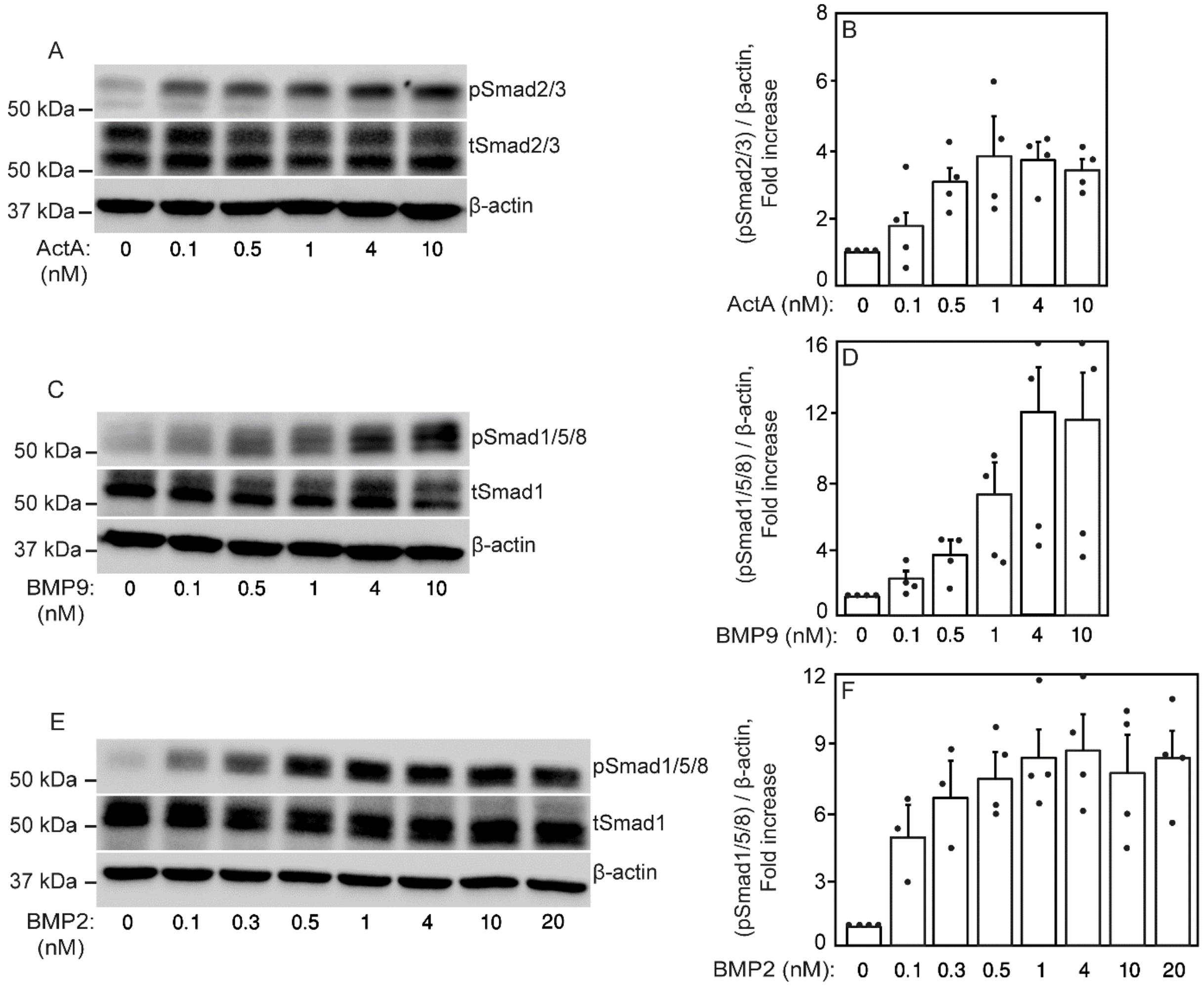

Fig. S2 Concentration dependence of Smad activation by ActA, BMP9 or BMP2 in U2OS cells. Cells were starved (2 h, 1% serum) and stimulated (30 min, 37 °C) with the indicated concentrations of ActA, BMP9 or BMP2, or left unstimulated in starvation medium (0 nM). Cells were lysed, subjected to SDS-PAGE and immunoblotted for pSmad2/3, tSmad2/3, and β-actin (for ActA stimulation) or for pSmad1/5/8, tSmad1 and β-actin (for BMP9 or BMP2). **a, c, e** Representative blots of stimulation by ActA (a), BMP9 (c), or BMP2 (e). **b, d, f** Quantification of ActA signaling to Smad2/3 (b), BMP9 signaling to Smad1/5/8 (d), and BMP2 signaling to Smad1/5/8 (f). The bands were visualized by ECL and quantified by densitometry (see Methods). Data are mean ± SEM (4 independent experiments in each case) of the ratio of pSmad2/3 (or pSmad1/5/8) over β-actin. The control values (0 nM) for stimulation with each ligand were taken as 1. In all cases, signaling increased with the ligand concentration up to saturation.

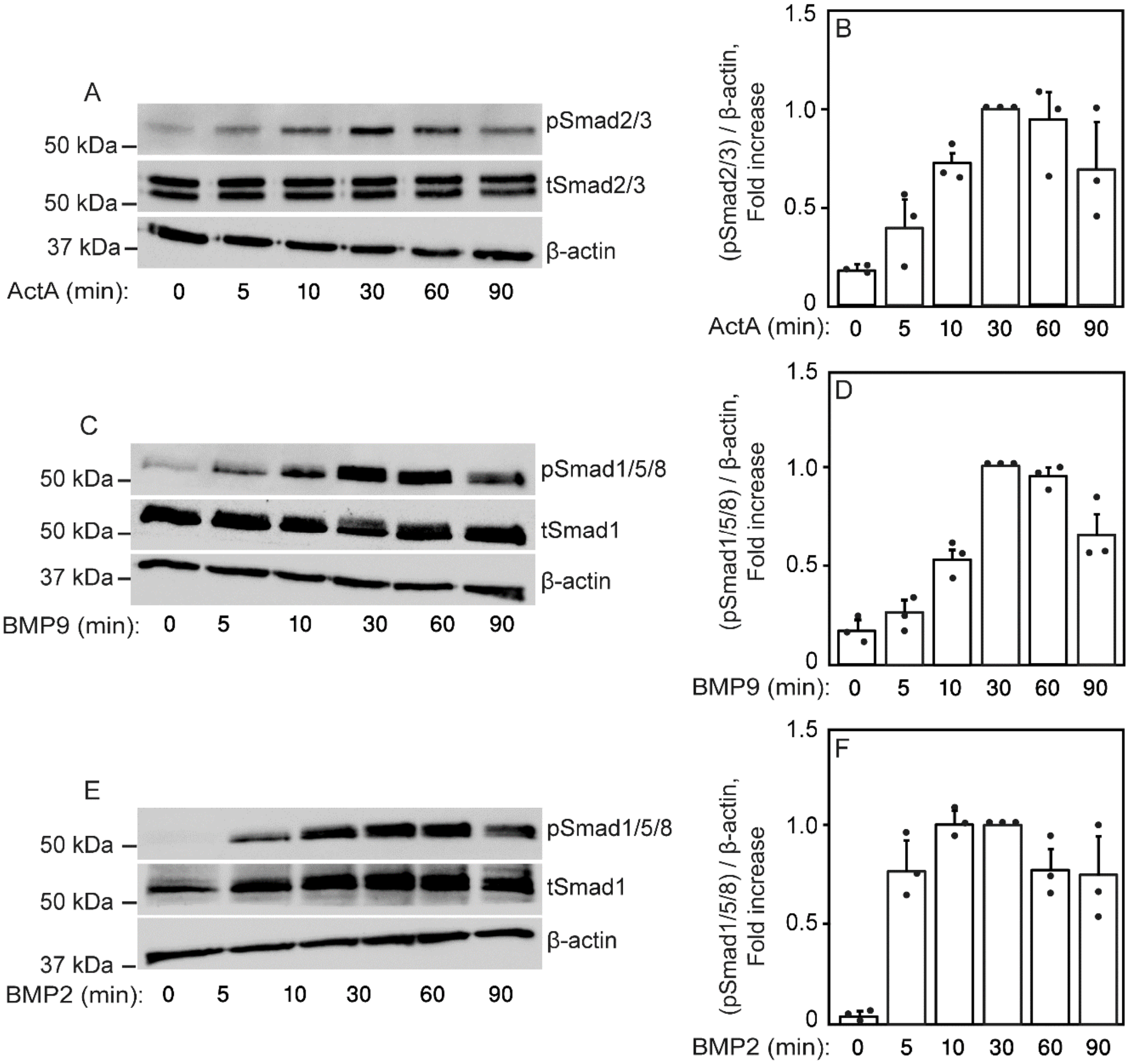

Fig. S3 Smad activation as a function of time by ActA, BMP9 or BMP2 in U2OS cells. Experiments were conducted as in Fig. S2, except that a single saturating concentration of each ligand was used (4 nM for ActA and BMP9, 10 nM for BMP2), and stimulation was for varying times (from 0 to 90 min). **a, c, e** Representative blots of stimulation by ActA (a), BMP9 (c), or BMP2 (e). **b, d, f** Quantification of ActA signaling to Smad2/3 (b), BMP9 signaling to Smad1/5/8 (d), and BMP2 signaling to Smad1/5/8 (f). The bands were visualized by ECL and quantified by densitometry (see Methods). Data are mean ± SEM (3 independent experiments in each case) of the ratio of pSmad2/3 (or pSmad1/5/8) over β-actin. The values obtained for 30 min stimulation with each ligand (which were the highest in all cases) were taken as 1.

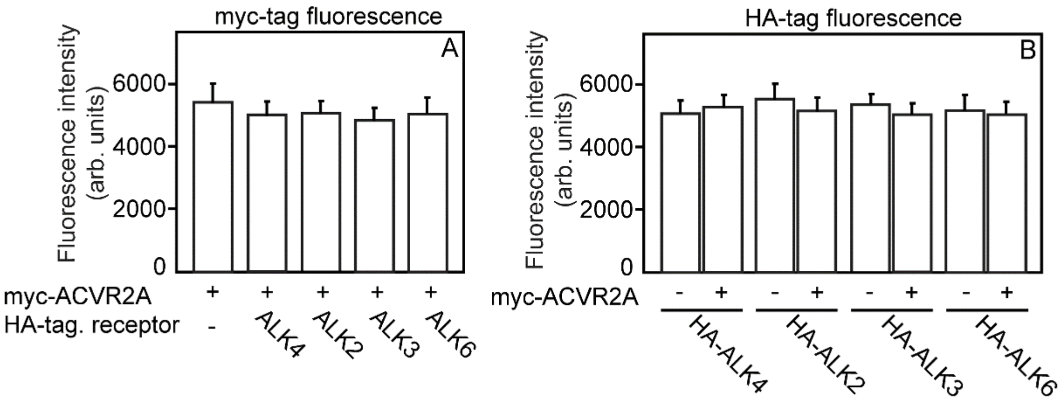

Fig. S4 myc-ACVR2A cell surface levels are unaffected by coexpression of HA-type I receptors, and vice versa. COS7 cells were transfected as in Fig. 2 by myc-ACVR2A alone or together with an HA-tagged type I receptor (or empty vector). After 24 h, the cell surface levels of the indicated receptors were measured by the point confocal method, as described under Methods and in Fig. 1a, using the FRAP setup under identical non-bleaching conditions. Results are mean ± SEM of 30 independent measurements (each on a different cell) under each condition. **a** Level of myc-ACVR2A alone or in the presence of HA-tagged type I receptors. **b** Level of HA-type I receptors expressed alone or together with myc-ACVR2A. No significant differences were found between the expression levels of singly-expressed myc-ACVR2A or the same receptor coexpressed with HA-tagged ALK2/3/4/6. Similarly, coexpression of myc-ACVR2A with any of the HA-tagged receptors did not alter the level of the HA-tagged receptor (one-way ANOVA and Bonferroni post-hoc test; $P > 0.1$).

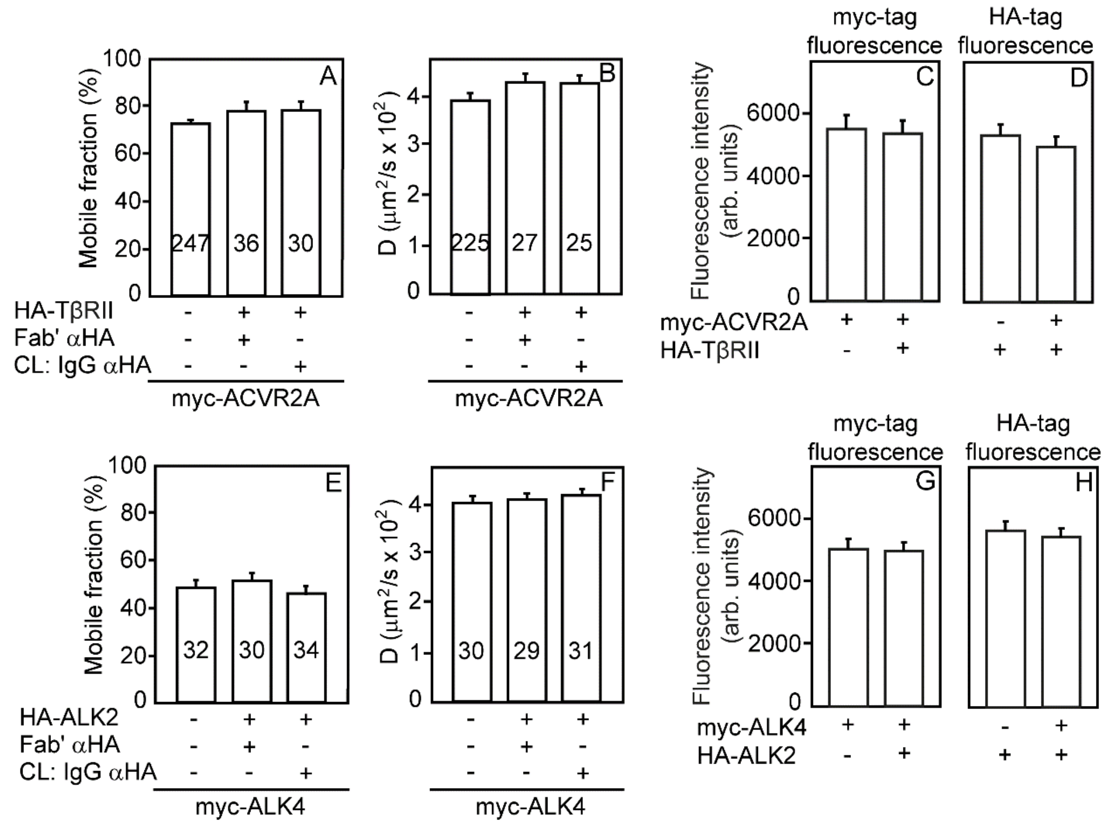

Fig. S5 Patch/FRAP studies do not detect interactions between myc-ACVR2A/HA-TβRII or myc-ALK4/HA-ALK2. COS7 cells were cotransfected with myc-ACVR2A and HA-TβRII or empty vector (a-d), or with myc-ALK4 and HA-ALK2 or empty vector (e-h). After 24 h, live cells were subjected to the IgG-mediated patching protocol (panels a, b, e, f) as in Fig. 2, resulting in the HA-tagged receptor patched and labeled by Alexa 488-GαR IgG (IgG αHA), whereas the myc-tagged receptor is labeled by monovalent Alexa 546-GαM Fab'. In control experiments, the IgG αHA labeling was replaced by Fab' αHA labeling. FRAP studies were conducted as in Fig. 2. **a, b** Average R_f (a) and *D* values (b) show no effect of crosslinking HA-TβRII on R_f or *D* of myc-ACVR2A. Bars depict the average values (mean ± SEM); the number of measurements (each conducted on a different cell) is shown on each bar. No significant differences were detected between the R_f or *D* values ($P > 0.29$; one-way ANOVA and Bonferroni post-hoc test). **c, d** The cell surface levels of myc-ACVR2A and HA-TβRII alone or together are similar (Student's two-tailed *t*-test; $P > 0.3$), as measured by the point confocal method (see Methods and Fig. 1a). Results are mean ± SEM of 30 independent measurements (each on a different cell) under each condition. **e, f** Average R_f (e) and *D* values (f) showing no effect of crosslinking HA-ALK2 on R_f or *D* of myc-ALK4. Bars, mean ± SEM values, with the number of measurements shown on each bar. No significant differences were found between the R_f or *D* values under all conditions ($P > 0.6$; one-way ANOVA and Bonferroni post-hoc test). **g, h** The cell surface levels of myc-ALK4 and HA-ALK2 alone or together are similar (Student's two-tailed *t*-test; $P > 0.8$); measurements were as in panels c, d.

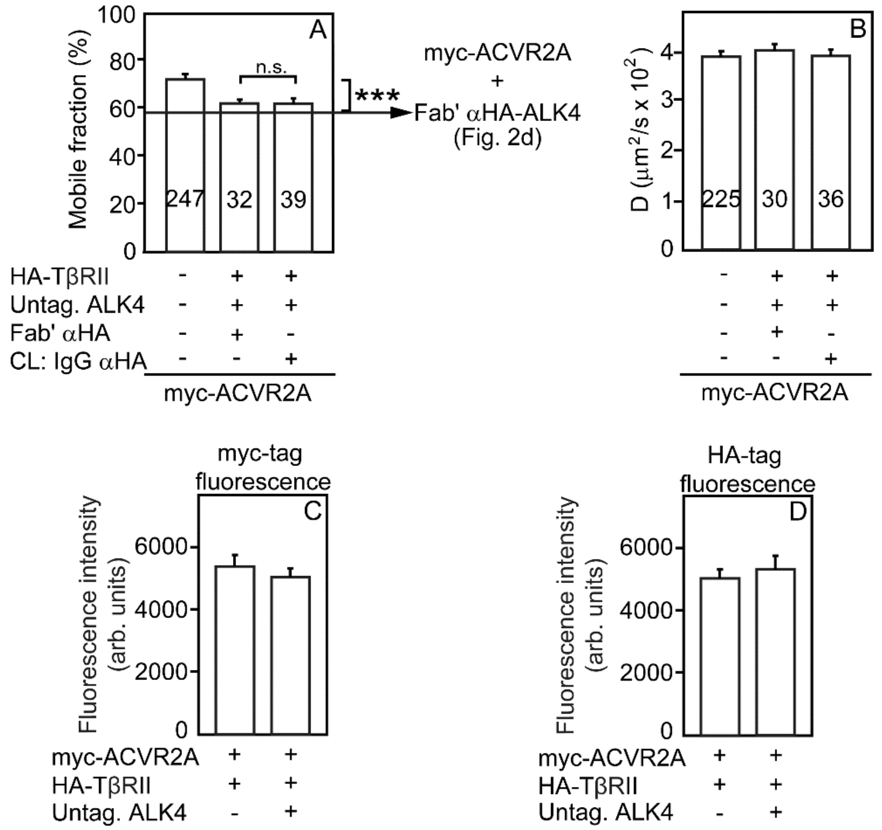

Fig. S6 HA-TβRII does not compete with ALK4 for binding myc-ACVR2A. Patch/FRAP experiments were conducted as in Figs. 3 and 4, on cells expressing myc-ACVR2A alone or together with HA-TβRII and untagged ALK4. **a** Average R_f values; **b** Average *D* values. Bars are mean ± SEM; the number of measurements is depicted on each bar. The full arrow shows the R_f values of myc-ACVR2A coexpressed with HA-ALK4 without IgG αHA crosslinking (taken from Fig. 2d, second bar from the left, as indicated to the right of the panel). No significant differences were found between the *D* values (panel b; one way ANOVA with Bonferroni post-hoc test, $P > 0.9$). Coexpression of HA-TβRII with ALK4 and myc-ACVR2A did not alter the reduction in R_f of the latter as measured upon coexpression of myc-ACVR2A with ALK4 alone (full line arrow; ***, $P < 10^{-4}$, one way ANOVA and Bonferroni post-hoc test), and this reduction was unaffected by IgG crosslinking of HA-TβRII (n.s. = non significant; $P > 0.9$). This indicates that HA-TβRII is not competing with ALK4 for binding myc-ACVR2A. **c, d** Point confocal measurements of the cell surface levels of coexpressed myc-ACVR2A and HA-TβRII with or without coexpression of untagged ALK4. Bars represents mean ± SEM of 30 independent measurements. No significant differences were observed between the levels of any receptor pairs compared (Student's two-tailed *t*-test; $P > 0.4$).

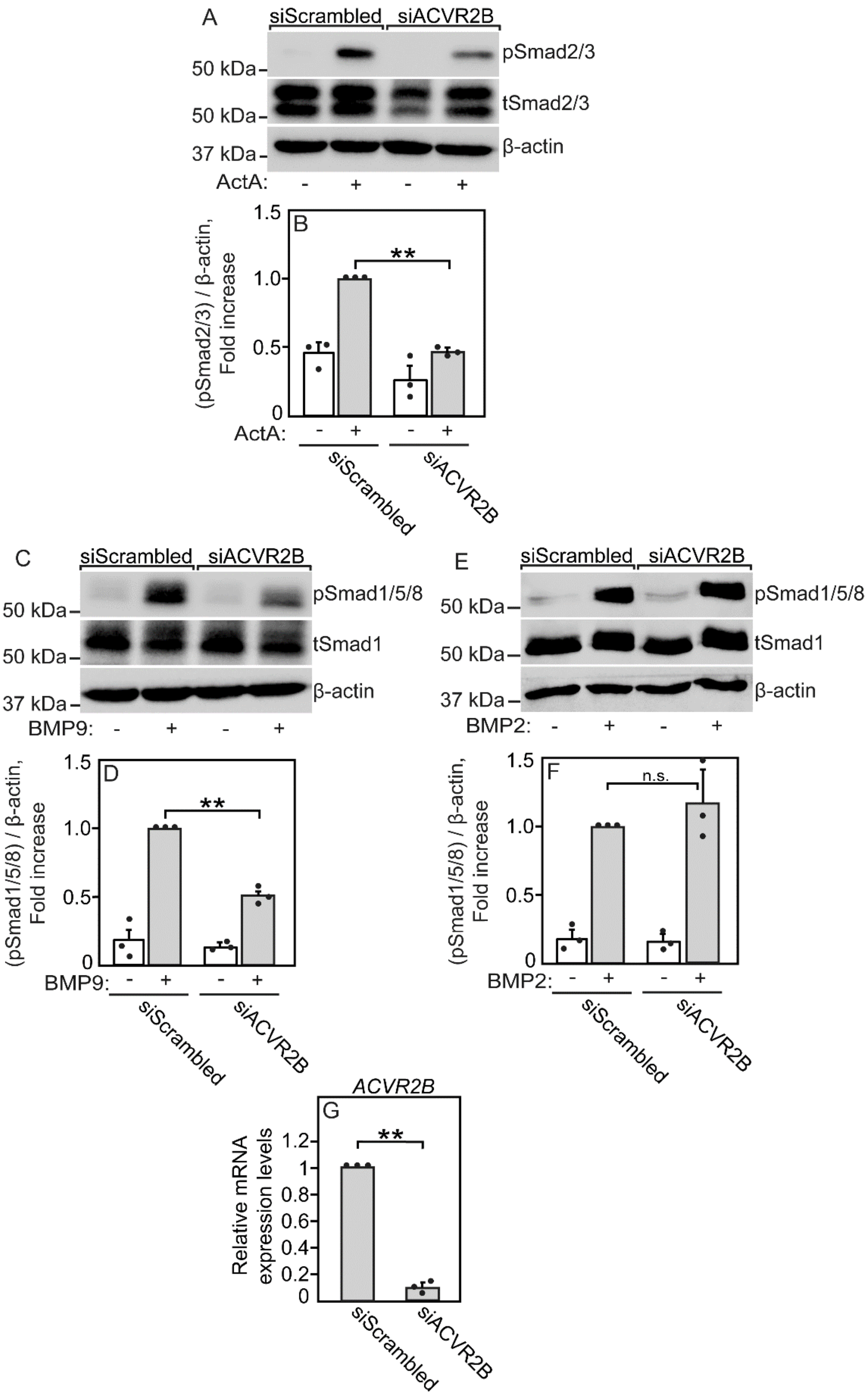

Fig. S7 ACVR2B signaling to Smad2/3 or Smad1/5/8 in U2OS cells. Experiments were as in Figs. 5 and 6, except that transfection was by siRNA to *ACVR2B*, with Scrambled siRNA as

control. After 24 h, cells were taken either for signaling (a-f) or RT-qPCR studies (g). For stimulation with ActA (4 nM, 30 min), the blots were probed for pSmad2/3, tSmad2/3 and β-actin. For BMP9 (4 nM) or BMP2 (10 nM) stimulation, they were probed for pSmad1/5/8, tSmad1 and β-actin. **a, c, e** Typical signaling experiments with ActA (a), BMP9 (c) or BMP2 (e). **b, d, f** Quantification of ActA signaling to Smad2/3 (b), and of BMP9 (d) or BMP2 (f) signaling to Smad1/5/8. Data are mean ± SEM of the pSmad/β-actin ratio of 3 independent experiments in each case. The value obtained for ligand-stimulated cells with siScrambled RNA was taken as 1. ActA signaling to pSmad2/3 and BMP9 signaling to pSmad1/5/8 were reduced by half after knockdown of *ACVR2B* (b, d), while BMP2 signaling to pSmad1/5/8 was unaffected (f). These data suggest that both ACVR2A (Figs. 5, 6) and ACVR2B can induce ActA signaling to Smad2/3 and BMP9 signaling to Smad1/5/8, while BMP2 signaling to Smad1/5/8 does not involve ACVR2B. **g** RT-qPCR showing the effect of siRNA to *ACVR2B* on its mRNA level. Data were normalized to *GAPDH,* taking *ACVR2B* mRNA level in siScrambled cells as 1. The transcript expression level of *ACVR2B* is given in Additional file 2: Table S1. Results are mean ± SEM of three independent experiments, each conducted in triplicate. Asterisks indicate significant differences between the bracketed pairs, using one-way ANOVA and Bonferroni post-hoc test for the signaling experiments (b, d and f; ***, $P < 10^{-4}$; n.s. = non significant, $P > 0.2$), and Student's two-tailed *t*-test for RT-qPCR (g; **, $P < 5 \times 10^{-4}$).

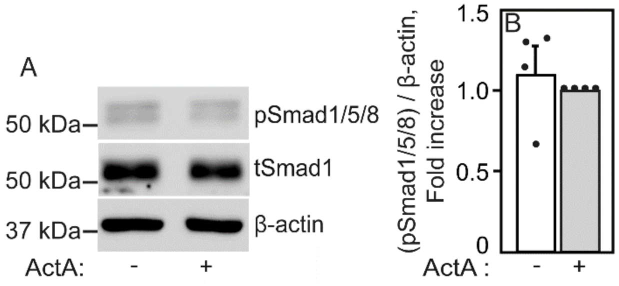

Fig. S8 ActA does not induce significant signaling to Smad1/5/8 in U2OS cells. Cells were starved (2 h, 1% serum) and stimulated (30 min, 37 °C) with 4 nM ActA or left in starvation medium (control). They were then lysed, subjected to SDS-PAGE and immunoblotted for pSmad1/5/8, tSmad1 and β-actin. **a** A blot of a representative experiment. **b** Quantification of ActA signaling to Smad1/5/8. The bands were quantified by ECL and densitometry. Data are mean ± SEM of the pSmad1/5/8 over β-actin ratio of 4 independent experiments. The value obtained for ActA-stimulated cells was taken as 1. No significant pSmad1/5/8 formation was detected following stimulation with ActA ($P > 0.5$; Student's two-tailed *t*-test).

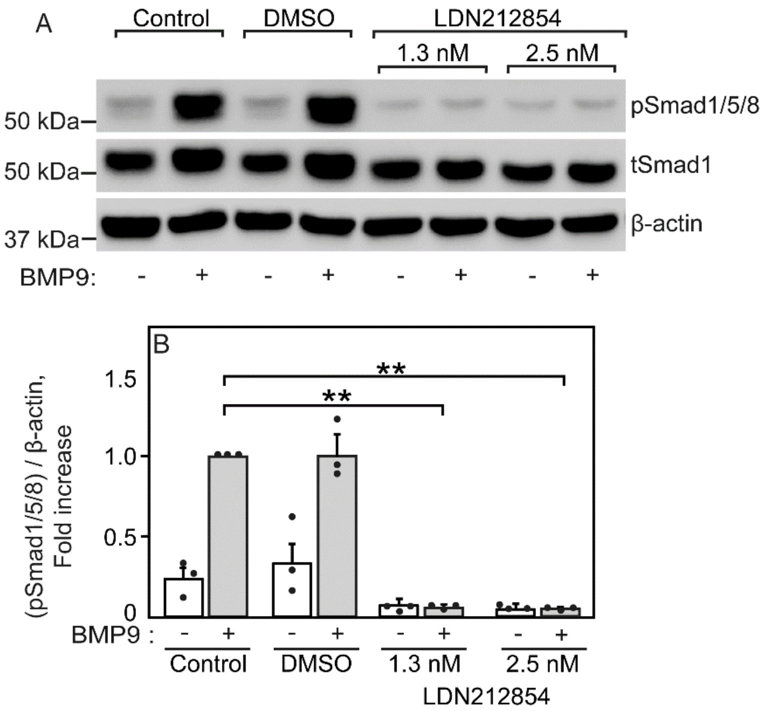

Fig. S9 The ALK2/3 inhibitor LDN212854 inhibits BMP9-mediated pSmad1/5/8 formation in U2OS cells. Cells were starved (2 h, 1% serum) and incubated for the second hour of starvation with LDN212854 (1.3 or 2.5 nM) or with 0.5% DMSO (vehicle). LDN212854 inhibits ALK2 and ALK1 (the latter of which is hardly expressed in U2OS cells; Additional file 2: Table S1) with IC50 of 1.3 and 2.4 nM, respectively, and ALK3 to a lower degree [67]. The incubation with the inhibitor was followed by stimulation (or not; control) for 30 min at 37 °C with BMP9 (4 nM). The cells were then lysed and immunoblotted for pSmad1/5/8, tSmad1 and β-actin as in Fig. 6. **a** A representative blot. **b** Quantification of BMP9 signaling to pSmad1/5/8. The bands were visualized by ECL and quantified by densitometry. Data are mean ± SEM of the pSmad1/5/8 over β-actin ratio of 3 independent experiments for each concentration. The value obtained for untreated cells stimulated with BMP9 was taken as 1. pSmad1/5/8 formation in response to BMP9 was strongly inhibited by LDN212854 at both 2.5 and 1.3 nM concentrations, suggesting that most of the signaling to pSmad1/5/8 in the U2OS cells is mediated *via* ALK2 with a possible contribution of ALK3. Asterisks indicate significant differences between the pairs marked by the brackets, using one-way ANOVA and Bonferroni post-hoc test (**, $P < 0.02$).

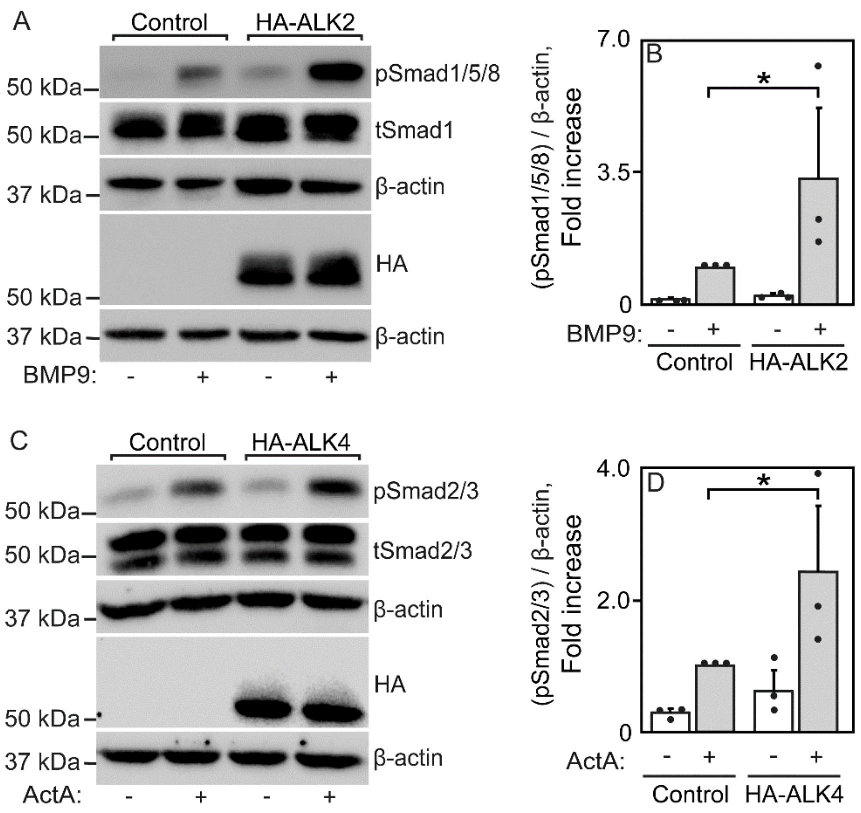

Fig. S10 Signaling activity of HA-ALK2 and HA-ALK4. To test whether HA-ALK2 and HA-ALK4 induce signaling, U2OS cells were transfected with HA-ALK2, HA-ALK4 or empty vector (control). After 24 h, cells were starved (2 h, 1% serum) and stimulated (30 min, 37 °C) with the indicated ligands (4 nM ActA or BMP9) or left in starvation medium. They were then lysed, subjected to SDS-PAGE and immunoblotted for pSmad1/5/8, tSmad1 (for BMP9 signaling), or for pSmad2/3 and tSmad2/3 (for ActA signaling); β-actin served as loading control. **a, c** Representative blots. **b, d** Quantification of BMP9 signaling to Smad1/5/8 by HA-ALK2 (b) and of ActA signaling to Smad2/3 by HA-ALK4 (d). The bands were quantified by ECL and densitometry. Data are mean ± SEM of the pSmad over β-actin ratio of 3 independent experiments in each case. The value obtained for ligand-stimulated control cells was taken as 1. In both cases, a significant increase was observed in the signaling following transfection with the respective HA-tagged receptor (one-way ANOVA and Bonferroni post-hoc test; *, $P < 0.05$).

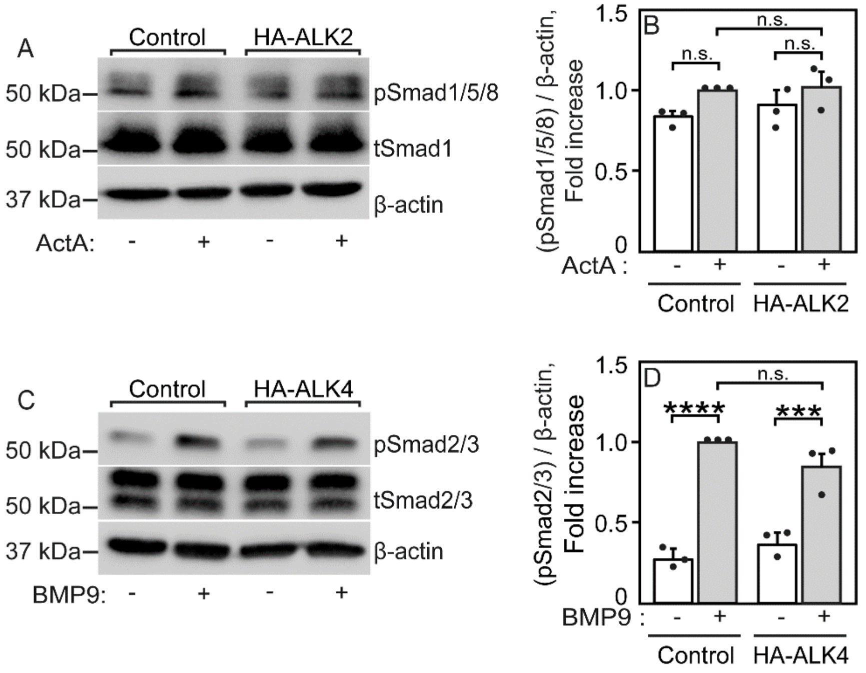

Fig. S11 HA-ALK2 and HA-ALK4 don't promote ActA signaling to Smad1/5/8 or BMP9 signaling to Smad2/3, respectively. To validate that overexpression of HA-ALK2 does not lead to ActA signaling to Smad1/5/8, and that overexpression of HA-ALK4 does not enhance BMP9-mediated signaling to Smad2/3, U2OS cells were transfected with either HA-ALK2, HA-ALK4 or empty vector (control). After 24 h, cells were starved (2 h, 1% serum) and stimulated (30 min, 37 °C) with the indicated ligands (4 nM ActA or BMP9), or left in starvation medium. They were then lysed, subjected to SDS-PAGE and immunoblotted for pSmad1/5/8, tSmad1 (for ActA signaling; panels a, b), or for pSmad2/3 and tSmad2/3 (for BMP9 signaling; panels c, d); β-actin served as loading control. **a, c** Representative blots. **b, d** Quantification of ActA signaling to Smad1/5/8 by HA-ALK2 (b) and of BMP9 signaling to Smad2/3 by HA-ALK4 (d). The bands were quantified by ECL and densitometry. Data are mean ± SEM of the pSmad over β-actin ratio of 3 independent experiments in each case. The value obtained for ligand-stimulated control cells was taken as 1. For ActA signaling to Smad1/5/8, no significant differences were obtained between any pair of samples indicated by the brackets, suggesting that overexpression of HA-ALK2 does not activate ActA signaling to Smad1/5/8 (one-way ANOVA and Bonferroni post-hoc test. n.s. = non significant; $P > 0.2$). Stimulation of pSmad2/3 formation by BMP9 could be detected (one-way ANOVA and Bonferroni post-hoc test; ****, $P < 10^{-4}$; ***, $P < 7 \times 10^{-4}$) in line with former studies [22-24], but overexpression of HA-ALK4 did not induce any further activation (n.s.; $P > 0.2$).

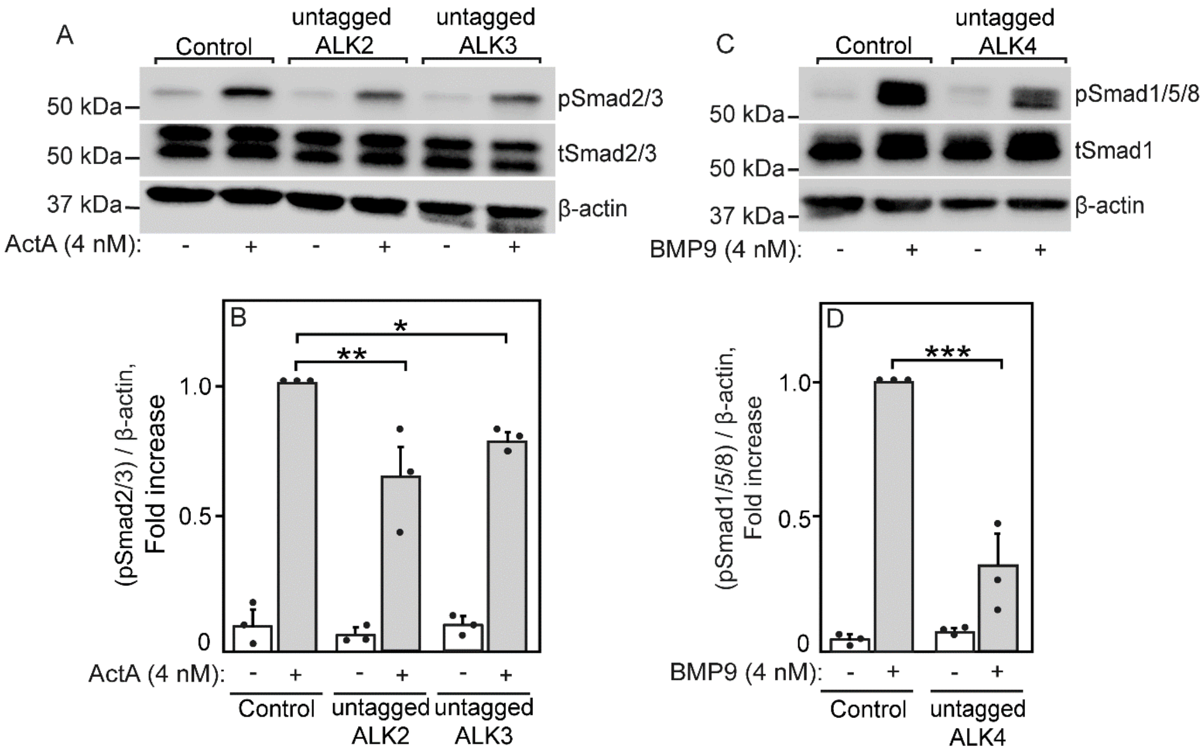

Fig. S12 Untagged ALK4 and type I BMP receptors compete for signaling to Smads *via* ACVR2. Experiments were done as in Fig. 8, except that the cells were transfected with the respective untagged receptors instead of the HA-tagged receptors. After activation with ActA (4 nM, 30 min) or BMP9 (4 nM, 30 min) the cells were lysed and immunoblotted for pSmad2/3, tSmad2/3 (for ActA signaling) and pSmad1/5/8, tSmad1 (for BMP9 signaling) along with β-actin. **a**, **c** Representative blots showing the effect of untagged ALK2 and ALK3 overexpression on ActA signaling to Smad2/3 (a) or of ALK4 overexpression on BMP9 signaling to pSmad1/5/8 (c). **b, d** Quantification of the effects of untagged ALK2 or ALK3 on ActA-mediated pSmad2/3 formation (b) and of untagged ALK4 on BMP9-mediated pSmad1/5/8 formation (d). Data are mean ± SEM of the relevant pSmad over β-actin ratio of 3 independent experiments in each case. The value obtained for control cells stimulated by the respective ligand was taken as 1. Asterisks indicate significant differences between the pairs marked by the brackets, using one-way ANOVA and Bonferroni post-hoc test (*, $P < 0.03$; **, $P < 8 \times 10^{-4}$; ***, $P < 10^{-4}$).

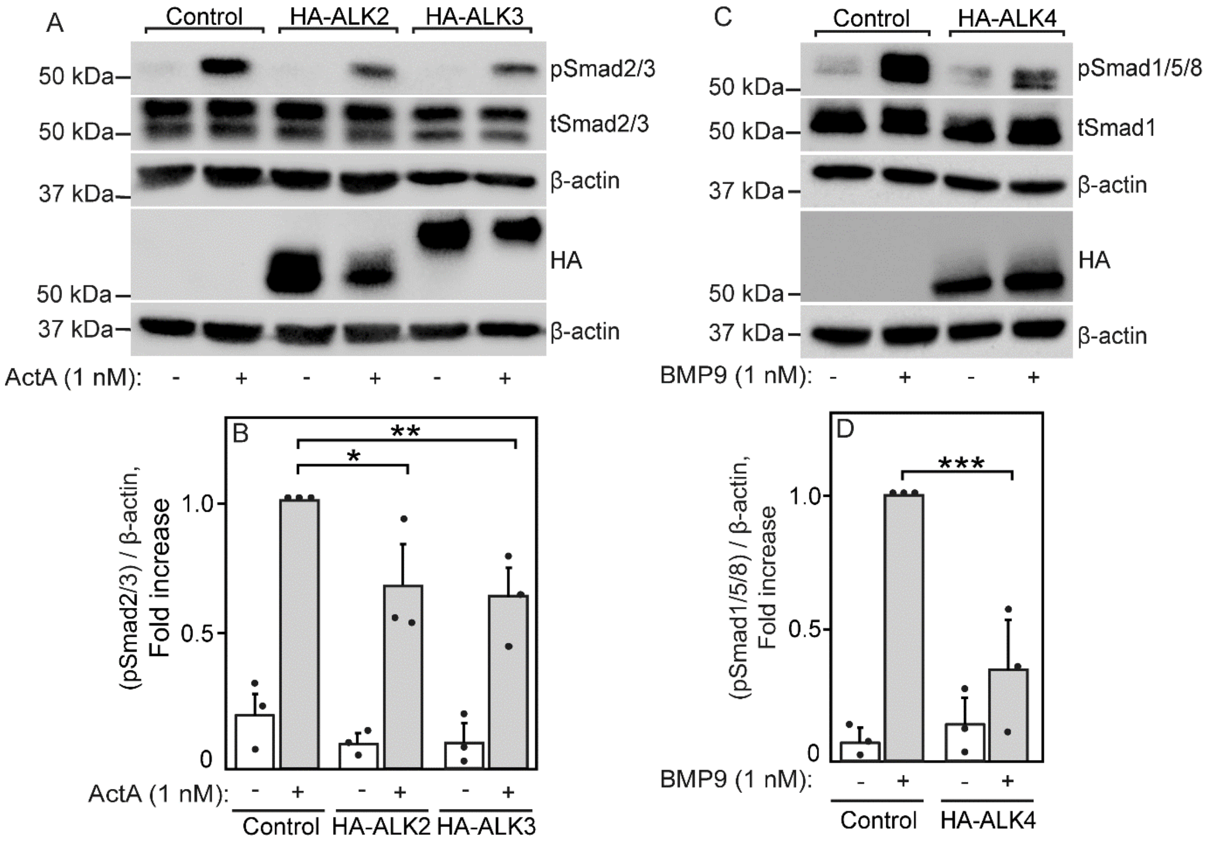

Fig. S13 Signaling competition by ALK4 and type I BMP receptors occurs also at lower ligand concentrations. Experiments were done as in Fig. 8, except that the ligand concentrations used for stimulation (30 min) were lowered from 4 nM to 1 nM. **a**, **c** Representative blots showing the effects of overexpressing HA-ALK2 or -ALK3 on ActA signaling to Smad2/3 (a), or of HA-ALK4 overexpression on BMP9 signaling to pSmad1/5/8 (b). The expression of the various HA-tagged receptors was probed by blotting for the HA tag using the HA-7 antibody (a, b). **b, d** Quantification of ALK2 or ALK3 effects on ActA-mediated pSmad2/3 formation (b) and of ALK4 on BMP9-mediated pSmad1/5/8 formation (d). Data are mean ± SEM of the relevant pSmad over β-actin ratio of 3 independent experiments in each case. The value obtained for control cells stimulated by the respective ligand was taken as 1. Asterisks indicate significant differences between the pairs marked by the brackets, using one-way ANOVA and Bonferroni post-hoc test (*, $P < 0.03$; **, $P < 0.01$; ***, $P < 5 \times 10^{-4}$).

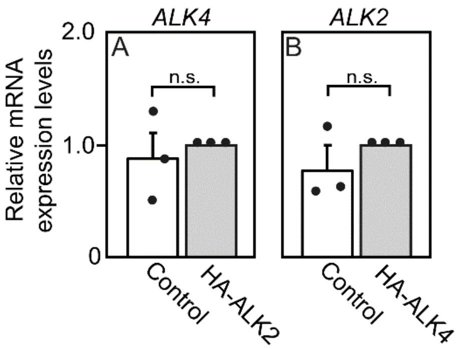

Fig. S14 Endogenous *ALK2* mRNA levels are not affected by overexpression of *ALK4* and *vice versa*. U2OS cells were transfected with empty vector (control), HA-ALK2 or HA-ALK4 as indicated at the bottom of the panels. At 24 h post-transfection, cells were taken for RT-qPCR determination of the mRNA levels of endogenous *ALK4* (a) or *ALK2* (b) as described under Methods. **a, b** Quantification of endogenous *ALK4* mRNA levels without and with overexpression of HA-ALK2 (a) and of endogenous *ALK2* mRNA levels without and with overexpression of HA-ALK4 (b). Data are mean ± SEM of the relevant mRNA levels of 3 independent experiments in each case. The value obtained for the cells transfected with the respective HA-tagged receptor was taken as 1. In both cases, no significant differences were observed between the control and the transfected samples (Student's two-tailed *t*-test. n.s. = non significant; $P > 0.3$).
