# Supplementary material for: Competition between type I activin and BMP receptors for binding to ACVR2A regulates signaling to distinct Smad pathways
Source: BMC Biol. 2022 Feb 18;20:50. doi: 10.1186/s12915-022-01252-z (PMC8855587; doi:10.1186/s12915-022-01252-z)
Supplement: Supplementary file 2 — Additional file 2: Table S1. Expression of TGF-β superfamily receptors in U2OS cells. Table S2. Sequences of the primer pairs used for RT-qPCR of each receptor gene. [file 12915_2022_1252_MOESM2_ESM.docx]

**Additional file 2: Supplementary Tables S1 and S2**

**Table S1 Expression of TGF-β superfamily receptors in U2OS cells.**

| **Gene name** | ***ACVR2A***  **(*ActRIIA*)** | ***ACVR2B***  **(*ActRIIB*)** | ***BMPR1A***  **(*ALK3*)** | ***BMPR1B***  **(*ALK6*)** | ***ACVR1***  **(*ALK2*)** | ***ACVR1B***  **(*ALK4*)** |
| --- | --- | --- | --- | --- | --- | --- |
| RPKM | 2.8157 | 3.56561 | 3.90216 | 1.21011 | 8.06852 | 10.55128 |
| **Gene name** | ***ACVR1C***  **(*ALK7*)** | ***ACVRL1***  **(*ALK1*)** | ***BMPR2***  **(*BMPRII*)** | ***TGFBR1***  **(*ALK5*)** | ***TGFBR2***  **(*TGFBRII*)** |  |
| RPKM | 0.06037 | 0.06102 | 11.54662 | 10.84656 | 22.66075 |  |

RPKM values (Reads Per Kilobase of transcript per Million mapped reads) were taken from the Cancer Cell Line Encyclopedia [[65](#_ENREF_64)], employing cBioPortal [[66](#_ENREF_65)].

**Table S2 Sequences of the primer pairs used for RT-qPCR of each receptor gene.**

| **Gene** | **Forward primer (5’ to 3’)** | **Reverse primer (5’ to 3’)** |
| --- | --- | --- |
| *ACVR2A* | CCTCCTGTACTTGTTCCAACTC | GCTTTCCAGACACAACCAAATC |
| *BMPRII* | GGCTGAACTTATGATGATTTGGGAA | CACGCCTATTATGTGACAGGTTGC |
| *ACVR2B* | Human *ACVR2B* qPCR Primer Pair Cat. #HP100284 (Sino Biological, Wayne, PA) | |
| *ALK2* | Human *ACVR1* qPCR Primer Pair Cat. #HP100282 (Sino Biological, Wayne, PA) | |
| *ALK3* | TTCGTATGACGGATCACTCG | AGCCCTACATCATGGCTGAC |
| *ALK4* | GTTCCTCCTGTTCCTCATCATC | GATCTTCCATGTCCAGTCTCTG |
| *ALK6* | GCTGCAGTTCCCTTGAGTTA | CTCTCTTCCTCCGTTGTCTTTC |
| *GAPDH* | CGGAGTCAACGGATTTGGTC | GAATTTGCCATGGGTGGAAT |
