# Supplementary material for: Competition between type I activin and BMP receptors for binding to ACVR2A regulates signaling to distinct Smad pathways
Source: BMC Biol. 2022 Feb 18;20:50. doi: 10.1186/s12915-022-01252-z (PMC8855587; doi:10.1186/s12915-022-01252-z)

Additional file 3: Original uncropped Western blots of all signaling experiments

Prior to probing the blots by the indicated antibodies, the membranes were cut in order to allow parallel processing of the different antigens with the respective antibodies.

Fig. 5A

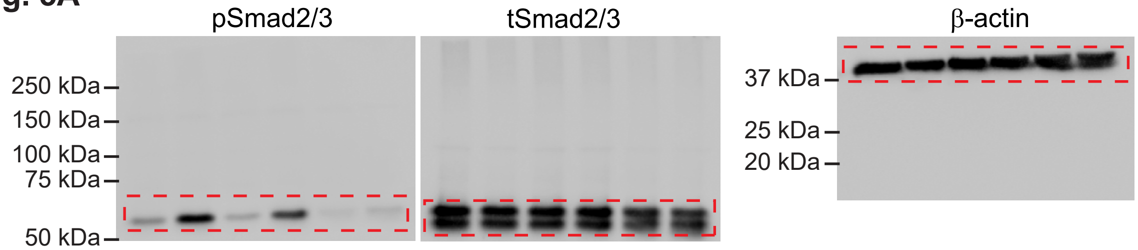

Fig. 6A

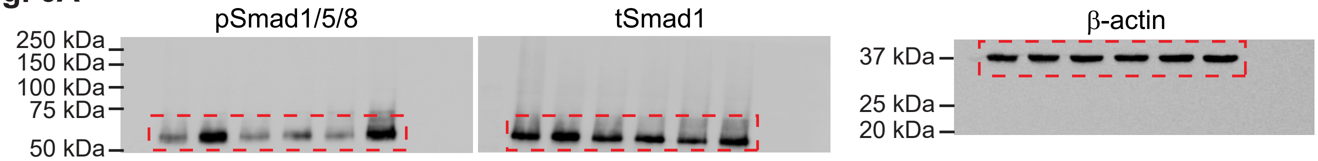

Fig. 6C

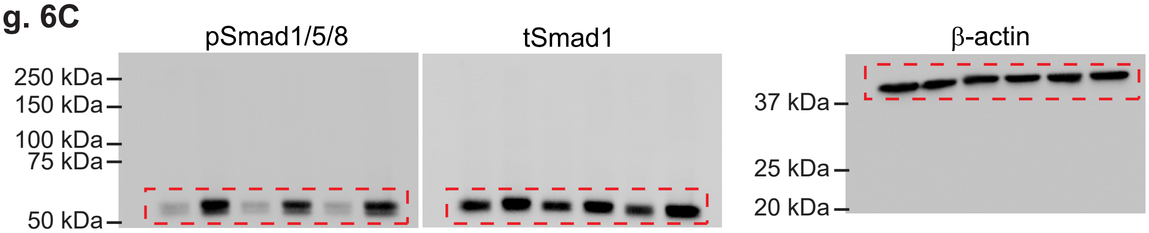

Fig. 7A

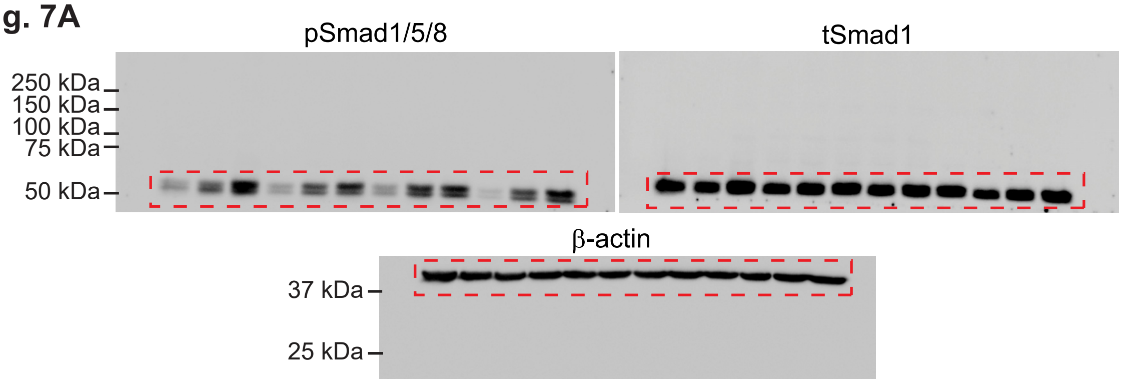

Fig. 8A

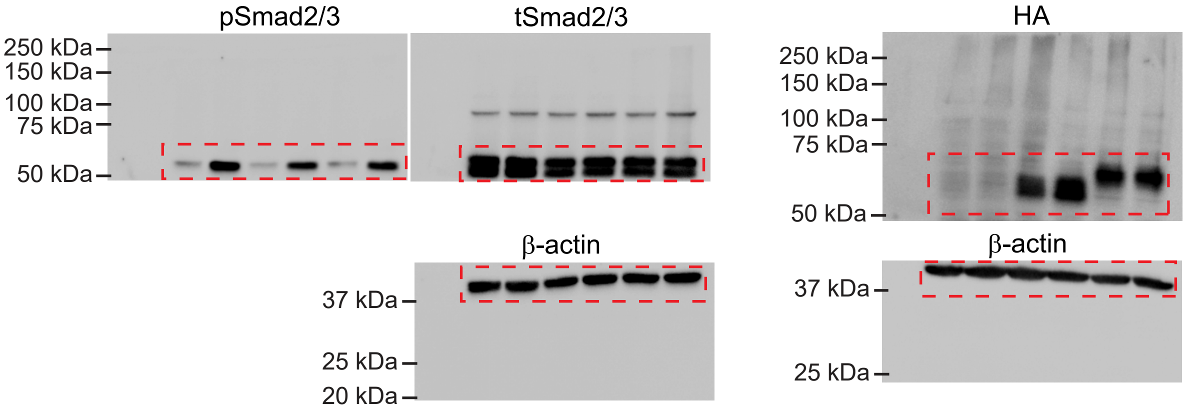

Fig. 8B

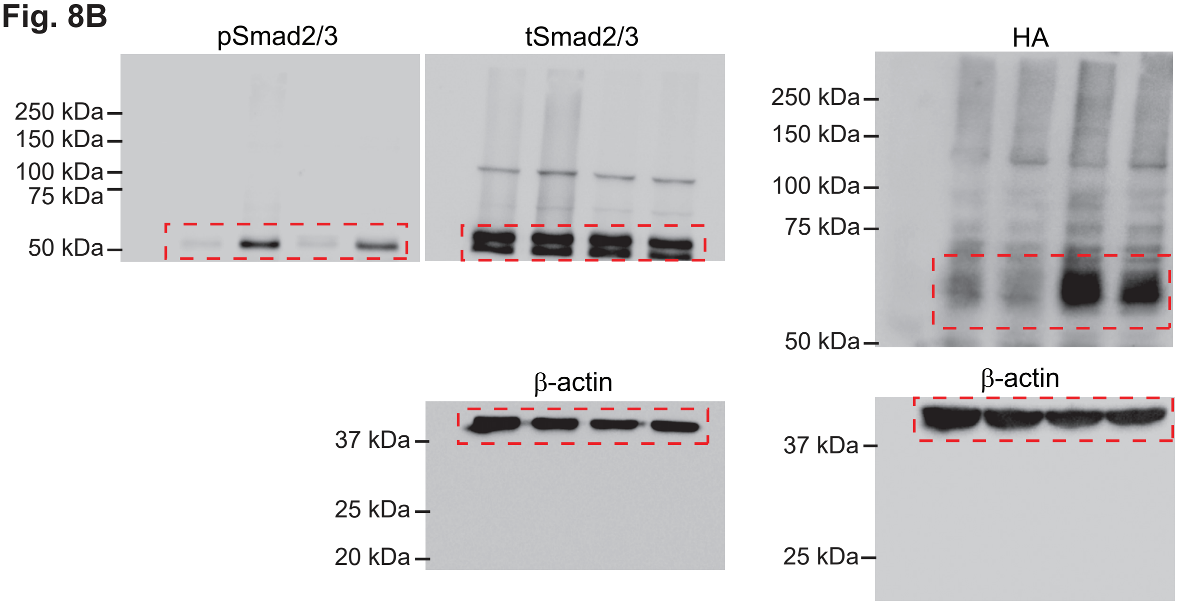

Fig. 8C

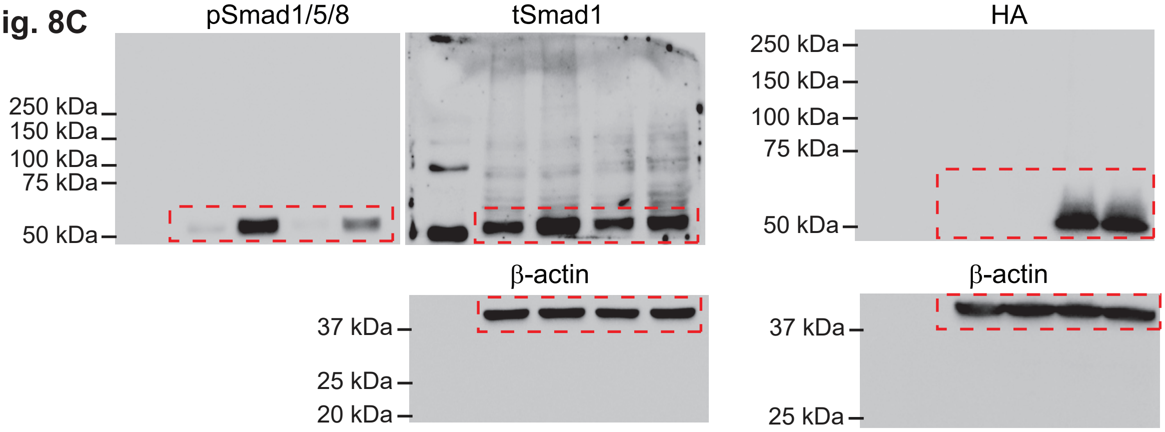

Fig. S2A

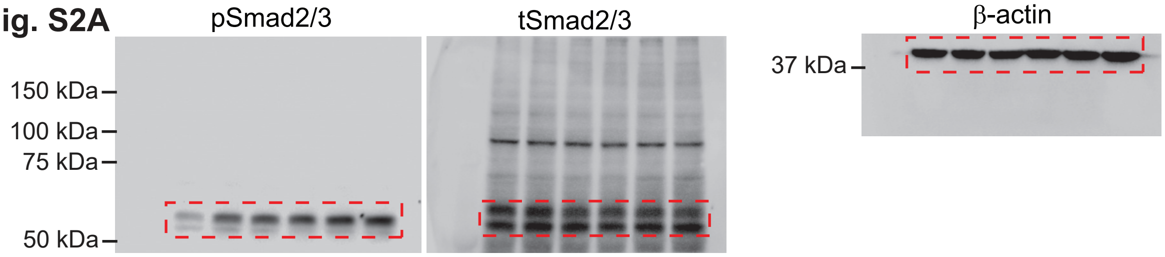

Fig. S2C

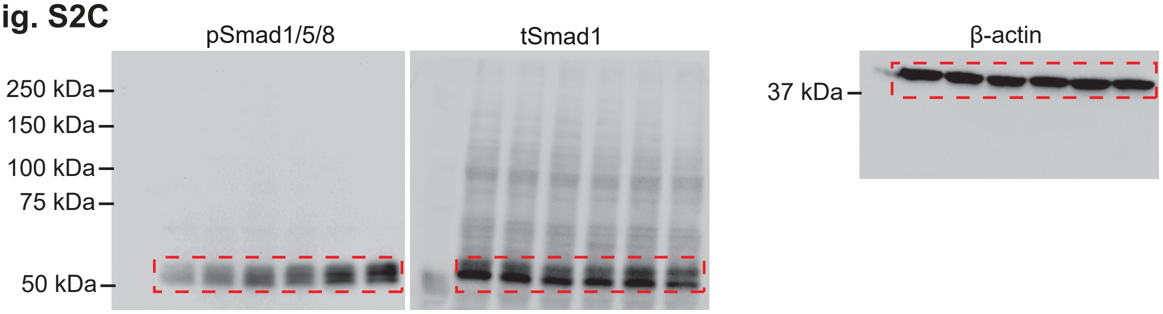

Fig. S2E

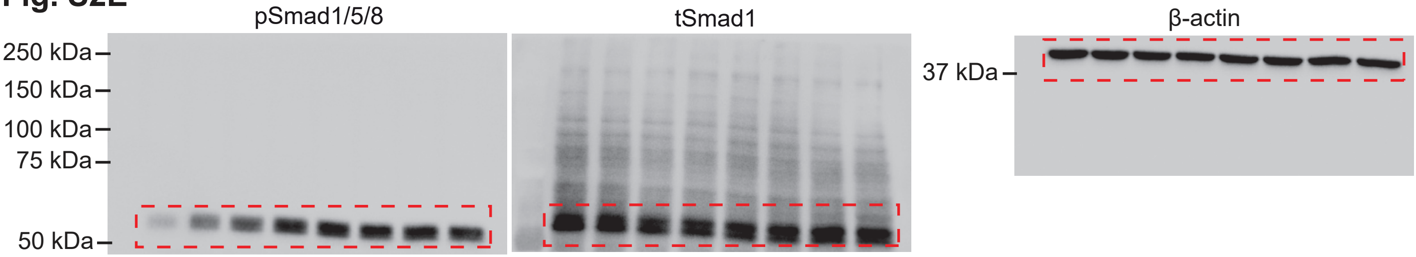

Fig. S3A

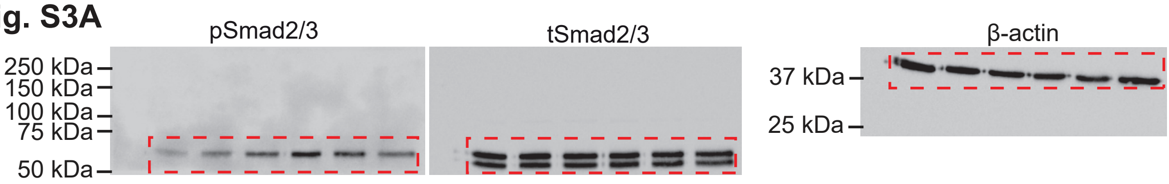

Fig. S3C

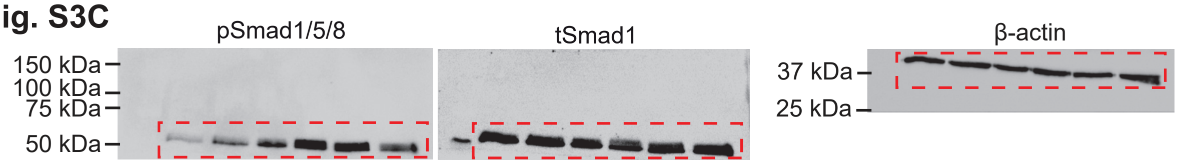

Fig. S3E

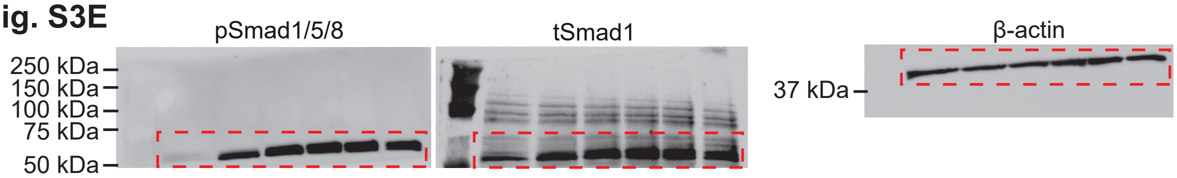

Fig. S7A

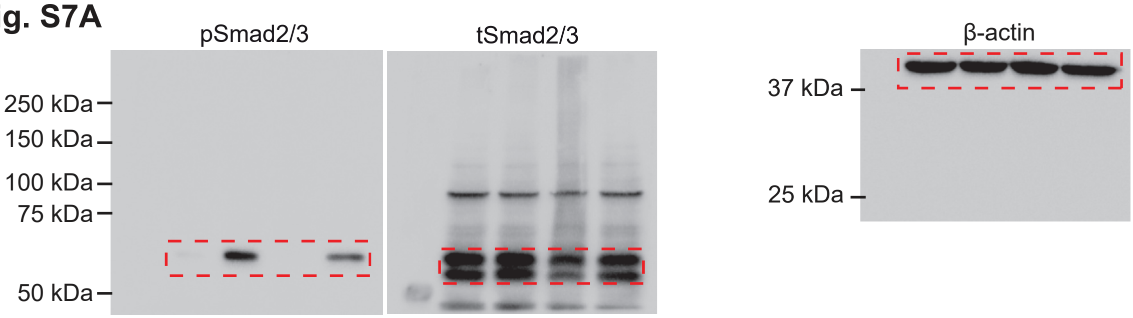

Fig. S7C

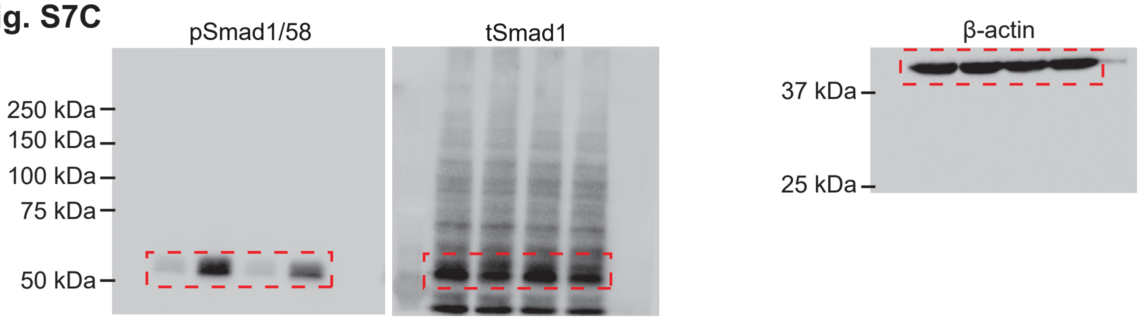

Fig. S7E

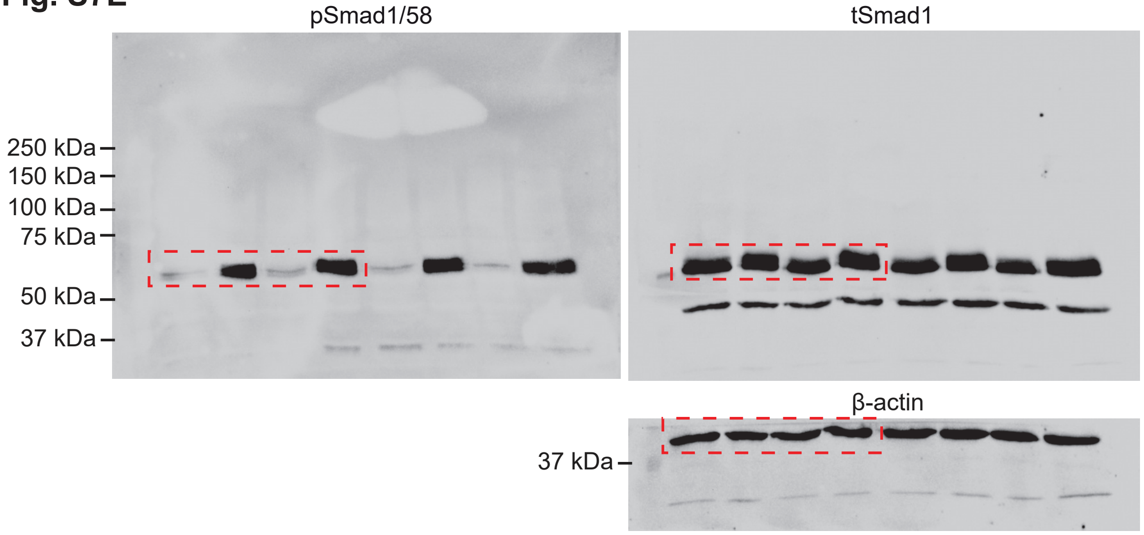

Fig. S8A

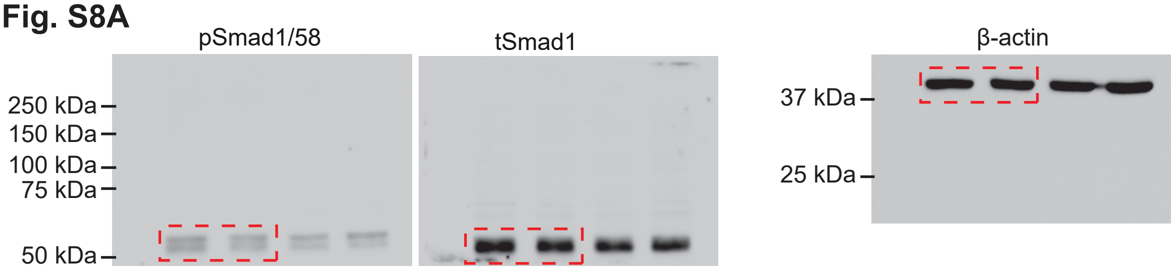

Fig. S9A

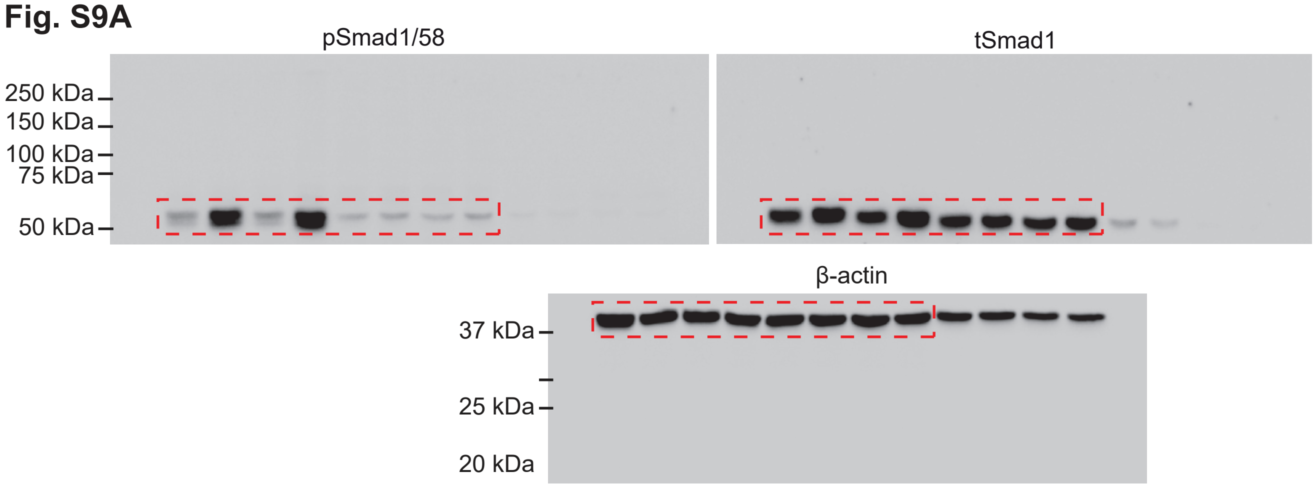

Fig. S10A

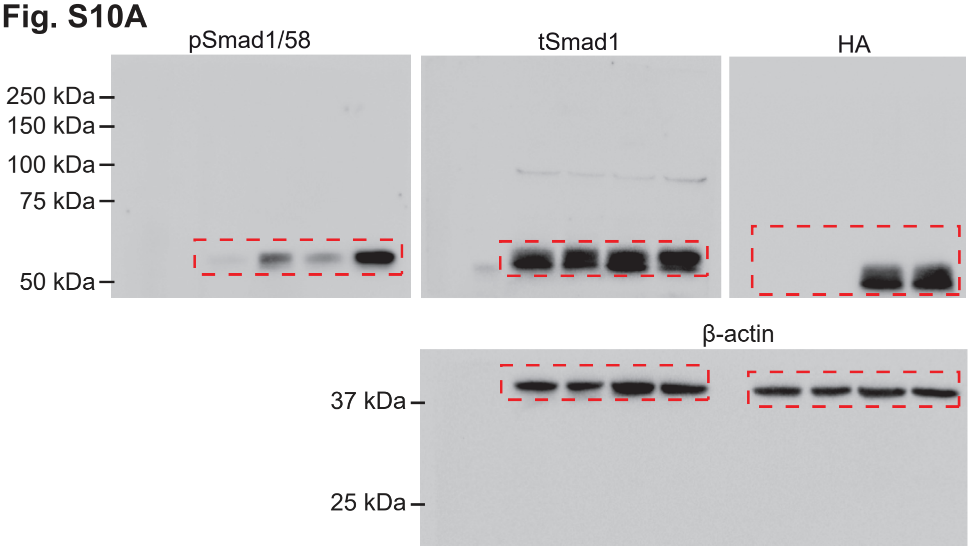

Fig. S10C

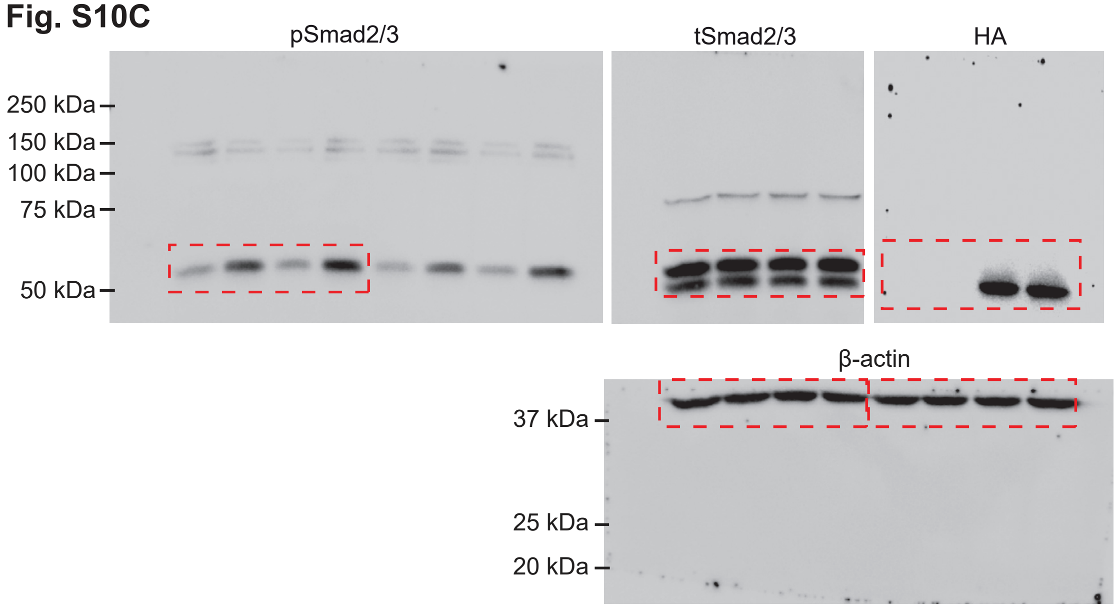

Fig. S11A

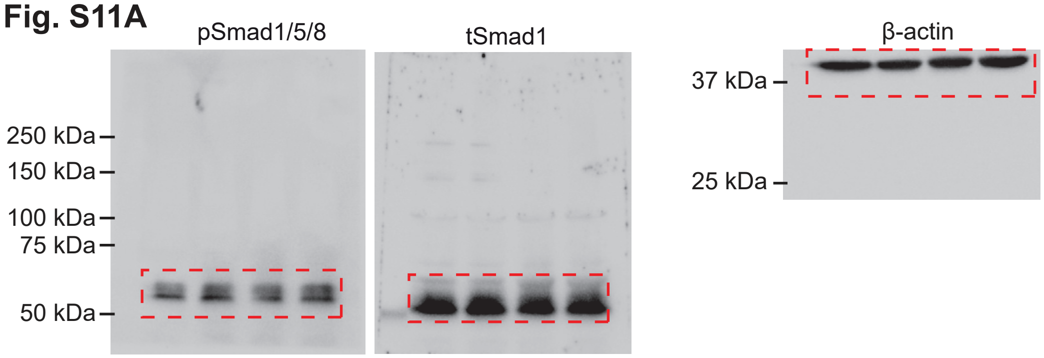

Fig. S11C

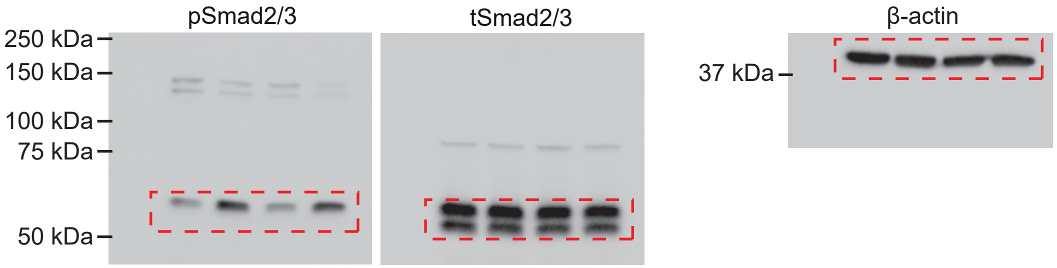

Fig. S12A

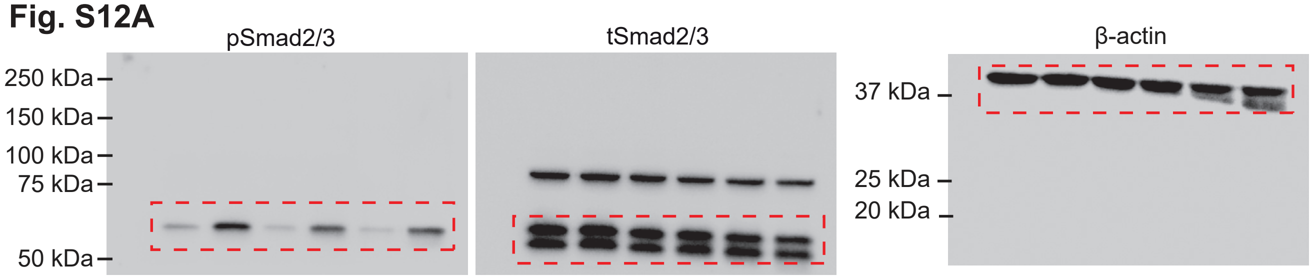

Fig. S12C

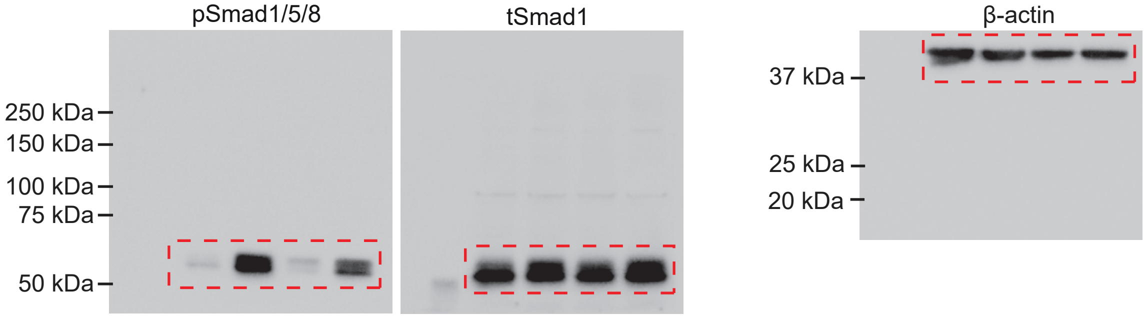

Fig. S13A

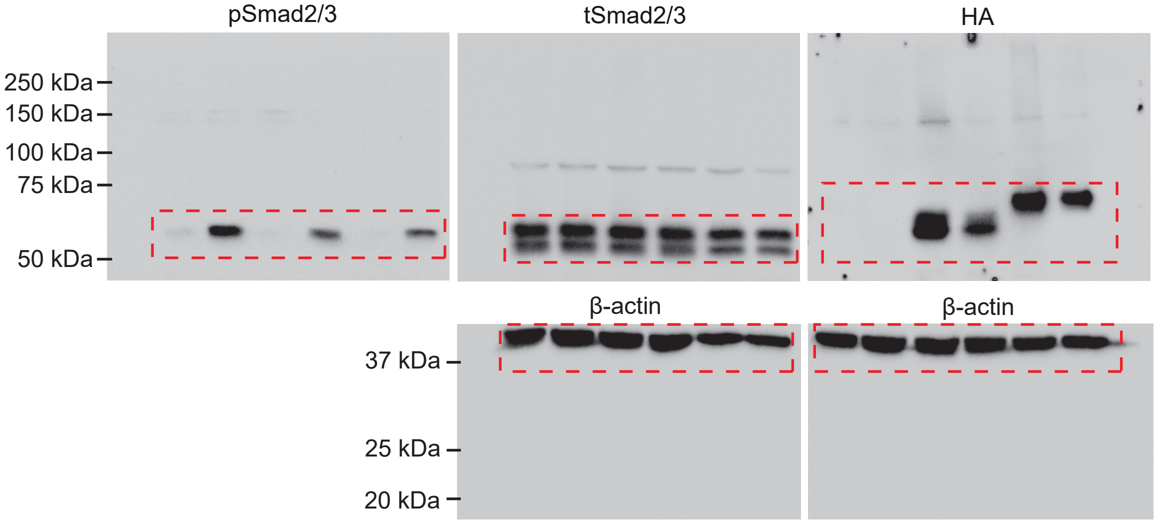

Fig. S13C

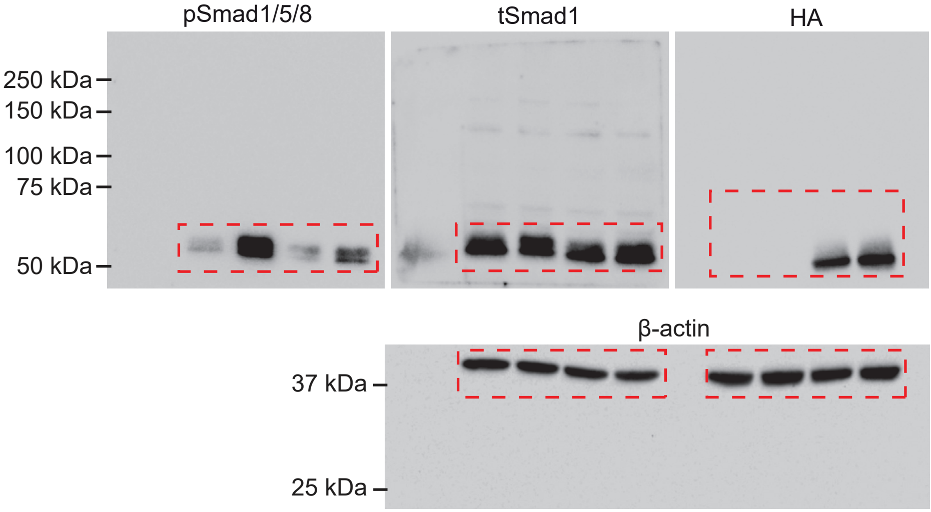

Supplement: Supplementary file 3 — Additional file 3. Original uncropped Western blots, PDF file. [file 12915_2022_1252_MOESM3_ESM.pdf]
